# Rice *ORMDL* Controls Sphingolipid Homeostasis Affecting Fertility Resulting from Abnormal Pollen Development

**DOI:** 10.1371/journal.pone.0106386

**Published:** 2014-09-05

**Authors:** Chutharat Chueasiri, Ketsuwan Chunthong, Keasinee Pitnjam, Sriprapai Chakhonkaen, Numphet Sangarwut, Kanidta Sangsawang, Malinee Suksangpanomrung, Louise V. Michaelson, Johnathan A. Napier, Amorntip Muangprom

**Affiliations:** 1 National Center for Genetic Engineering and Biotechnology, Thailand Science Park, Klong Luang, Pathumthani, Thailand; 2 Biological Chemistry Department, Rothamsted Research, Harpenden, Hertfordshire, United Kingdom; University College Dublin, Ireland

## Abstract

The orosomucoids (ORM) are ER-resisdent polypeptides encoded by *ORM* and *ORMDL* (ORM-like) genes. In humans, ORMDL3 was reported as genetic risk factor associated to asthma. In yeast, ORM proteins act as negative regulators of sphingolipid synthesis. Sphingolipids are important molecules regulating several processes including stress responses and apoptosis. However, the function of *ORM*/*ORMDL* genes in plants has not yet been reported. Previously, we found that temperature sensitive genetic male sterility (TGMS) rice lines controlled by *tms2* contain a deletion of about 70 kb in chromosome 7. We identified four genes expressed in panicles, including an *ORMDL* ortholog, as candidates for *tms2*. In this report, we quantified expression of the only two candidate genes normally expressed in anthers of wild type plants grown in controlled growth rooms for fertile and sterile conditions. We found that only the *ORMDL* gene (*LOC_Os07g26940*) showed differential expression under these conditions. To better understand the function of rice *ORMDL* genes, we generated RNAi transgenic rice plants suppressing either *LOC_Os07g26940*, or all three *ORMDL* genes present in rice. We found that the RNAi transgenic plants with low expression of either *LOC_Os07g26940* alone or all three *ORMDL* genes were sterile, having abnormal pollen morphology and staining. In addition, we found that both sphingolipid metabolism and expression of genes involved in sphingolipid synthesis were perturbed in the *tms2* mutant, analogous to the role of ORMs in yeast. Our results indicated that plant ORMDL proteins influence sphingolipid homeostasis, and deletion of this gene affected fertility resulting from abnormal pollen development.

## Introduction

Orosomucoid (ORM) family proteins are ER proteins encoded by *ORM* and *ORMDL* (ORM-like) genes. These proteins are highly conserved from yeasts to plants to human. Recently, human ORMDL3 was reported as a genetic risk factor associated with asthma in diverse populations [1]–[3]. In addition, the expression of *ORMDL3* gene was shown to be associated with the disease symptoms [4], [5]. ORMDL3 is involved in pro-inflammatory diseases by binding and inhibiting sarco-endoplasmic reticulum Ca^2+^ pump (SERCA), which reduce ER Ca^2+^ concentration and increase unfolded-protein response (UPR), a process believed to induce inflammation [6].

In yeast, deletion of the ORM proteins showed susceptibility to agents that increase protein-misfolding in the ER [7]. In addition, deletion of the ORM proteins increased UPR and slowed ER-to-Golgi transport of the tested proteins though these effects were suppressed by high temperatures [8]. After treating with agents that increase protein-misfolding, *ORM2* gene expression was increased significantly, indicating that it is up-regulated by UPR [8]. In addition, genetic interaction profiles of *orm2*Δ deletion mutants showed inverse correlation with the interaction patterns of yeast mutants with reduced *LCB1* and *LCB2* expression. Furthermore, yeast cells with over-expression of *ORM1* or *ORM2* showed genetic interaction profiles highly correlated with those seen in yeasts with reduced *LCB1* and *LCB2* expression, indicating that increased expression of ORM reduced LCB 1/2 activity [9]. *LCB1* and *LCB2* encode serine palmitoyltransferase, the first and rate-limiting enzyme in sphingolipid biosynthesis. Over-expression of *ORM1* or *ORM2* resulted in reduced LCB levels, while cells with deleted *ORM1/2* had highly elevated levels of LCBs [9]. Thus, it was proposed that ORM proteins were negative regulators of sphingolipid synthesis [9]. Yeast cells with *ORM1/2* deletions increased flux throughout the sphingolipid pathway, resulting in growth defects [9] though the precise metabolic impact of loss of ORM function on sphingolipid synthesis in yeast is currently ambiguous, since *orm1*Δ*/orm2*Δ mutants have been reported to have reduced [8] or elevated [9] levels of ceramides. In addition, alteration in *ORM* gene expression or mutations to their phosphorylation sites perturbed sphingolipid metabolism, leading to the hypothesis that ORMs play a central role in lipid homeostasis [9], [10].

Sphingolipids are key cellular membranes and signaling molecules involved in several cellular activities, such as cell proliferation, cell differentiation, apoptosis, and stress responses [11]–[14]. In plants, sphingolipids are important for cell growth and the establishment of cell polarity through their contribution to the functional organization of the endomembrane system [15]. Accordingly, sphingolipids were reported to be involved in a trafficking pathway with specific endomembrane compartments and polar auxin transport protein [16]. Sphingolipids are also reported to be involved in responses to ABA and cold temperature [17]-[19], also regulated mineral ion homeostasis and programmed cell death [20], [21].

Microsporogenesis has been reported to be controlled by sphingolipids in several plant species [22]–[24]. In Arabidopsis, the study of *fbr11-2*/*lcb1-1* mutants showed that the *fbr11-2* mutant, an allele of *lcb1-1*, was transmitted only through female gametophytes, and initiated apoptotic cell death in bi-nucleated microspores [23]. The *FBR11/LCB1* expression was confined in microspores during microgametogenesis. These results suggested that SPT modulated programmed cell death plays an important role in the regulation of male gametophyte development [23]. In addition, Arabidopsis *LCB2* loss-of-function mutant demonstrated that sphingolipids are important for gametophytic development by alterations in the endomembrane system of pollen [22]. Furthermore, sterile or severely reduced in fertility were observed in antisense or RNAi transgenic plants with repressed expression of *dihydrosphingosine C4 hydroxylase 1(DSH 1)*, a key constituent of sphingolipids, which supported the role of sphingolipids on fertility in plant [25].

Male sterility facilitates hybrid seed production. Rice hybrids showed 20% higher yields than the best inbred varieties [26]. Hybrid rice is a promising alternative to increase food production to meet the demand for future need. Previously, we found that *temperature-sensitive genetic male-sterile 2* (*tms2*) rice line (TGMS) lacked a 70Kb section of DNA that encoded 4 expressed genes, one of which is an *ORMDL* ortholog [27]. Although there are three *ORMDL* genes in rice, only one (*LOC_Os07g26940*) is located in the deleted region. In this report, we found that the *LOC_Os07g26940* gene showed strong expression in rice panicles and anthers. In addition, *LOC_Os07g26940* showed differential expression under low and high temperatures, corresponding to fertile and sterile conditions, respectively. When RNAi was used to suppress *ORMDL* genes, either in the deletion region or all *ORMDL* three genes in rice, the resulting transgenic plants showed abnormal pollen development that resulted in male sterility. In addition, we showed that sphingolipids metabolism and expression of genes in sphingolipids biosynthesis were perturbed in the *tms2* mutants. Similar to yeast, our results indicated that the plant *ORMDL* gene modulates sphingolipid homeostasis, affecting fertility by controlling pollen development. Therefore, understanding the processes that control plant fertility could be useful in generating high yield crops by hybrid or by genetic engineering.

## Results

### Expression analysis of candidate genes

**Figure 1:**
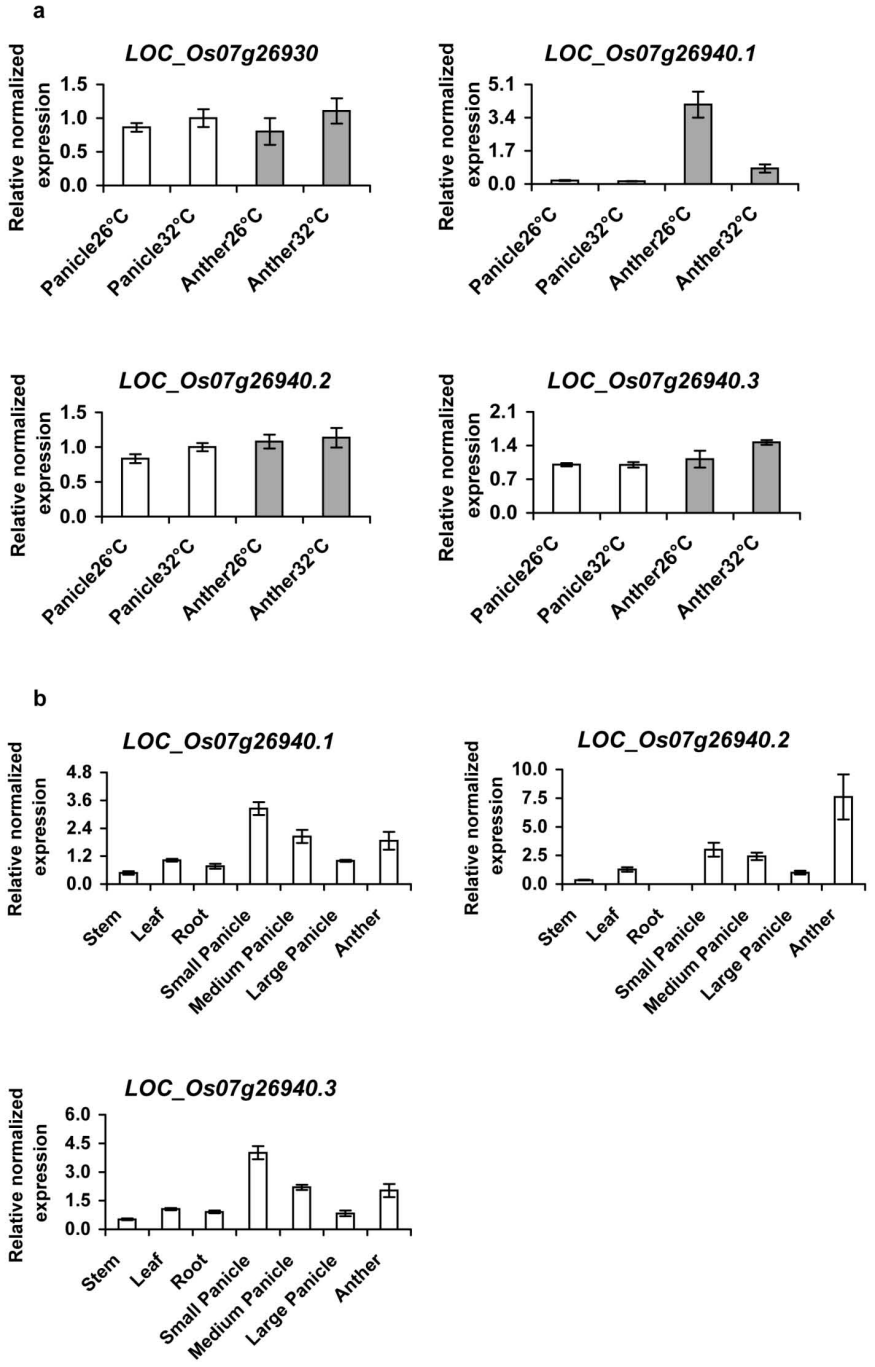
Expression analysis by quantitative RT-PCR. (A) qRT-PCR analysis of *LOC_Os07g26930* and all three splicing forms of *ORMDL LOC_Os07g26940* in panicles and anthers of wild type rice plants grown in control growth room at 26°C and 32°C. The expression level of *Actin1* (*Os05g36290*) was used as internal control. (B) qRT-PCR analysis of all three splicing forms of *LOC_Os07g26940* in wild type rice tissues, including stems, leaves, roots, small panicles (1–2 cm), medium panicles (3–14 cm), large panicles (15–25 cm) and anthers. The expression level of *EF-1α* (*Os03g08020*) was used as internal control. All data are representative from at least two biological repeats, each based on three technical replicates; similar results were obtained in the repeated experiments. Bars indicate the standard error (n = 3), which was calculated from technical replicates. Each biological sample was a mixture of 3 plants.

Seven genes located in the 70 Kb loci associated with the TGMS *tms2* phenotype were annotated to encode expressed proteins, four of which expressed in panicles [27]. In this study, we examined the expression of these four genes (*putative cytochrome P450 77A3 LOC_Os07g26870*, *LOC_Os07g26930*, *ORMDL LOC_Os07g26940*, and *LOC_Os 07g26974*) in anthers of wild type plants by RT-PCR, confirming the identity of the amplicons by sequencing. Our results indicated that only *LOC_Os07g26930* and *ORMDL LOC_Os07g26940* were expressed in anthers (data not shown). To determine whether *LOC_Os07g26930* and *ORMDL LOC_Os07g26940* were differentially expressed at low and high temperatures corresponding to fertile and sterile conditions, the expression of these genes was monitored in panicles and anthers of wild type plants grown in controlled growth room at 26 and 32°C, using qRT-PCR. Information from GRAMENE rice database indicated that LOC_Os07g26930 and *ORMDL LOC_Os07g26940* have 1 and 3 splicing forms respectively. Primers specific for all these forms were designed and used for expression analysis by qRT-PCR, compared to *Actin1* as internal control. The results showed that expression of *LOC_Os07g26930* was constant at low and high temperatures in panicle or in anther of wild type plants (Fig. 1A), inconsistent with a role in TGMS. In the case of *ORMDL LOC_Os07g26940*, we detected all three splicing forms by qRT-PCR. The results showed that expression levels of *ORMDL LOC_Os07g26940.1* was similar at low and high temperatures in the panicle. Interestingly in the anther, this splicing form showed much higher expression at low temperature (fertile condition) than that at high temperature (sterile condition) (Fig. 1A). *ORMDL LOC_Os07g26940.2* and *ORMDL LOC_Os07g26940.3* showed similar levels of expression at low and high temperatures in panicle or in anther, with a little higher at high temperatures in panicle for *ORMDL LOC_Os07g26940.2*. While *ORMDL LOC_Os07g26940.3* showed a little higher expression in the anther at high temperature (sterile condition) than at low temperature (fertile condition) (Fig. 1A). To further study tissue-specific expression of *ORMDL LOC_Os07g26940*, multiple different rice tissues were used for qRT-PCR analyses including leaves, stems, roots, anthers, small panicles, medium panicles, and large panicles. Using *elongation factor 1 α, EF-1α* as a reference gene, the results indicated that all the three transcripts derived from *ORMDL LOC_Os 07g26940* were detected in most of the tested tissues, except in root where we could not detect expression of *ORMDL LOC_Os 07g26940.2*. The three forms were predominantly accumulated in the panicle and anther (Fig. 1B).

### Phylogenetic Analysis

**Figure 2:**
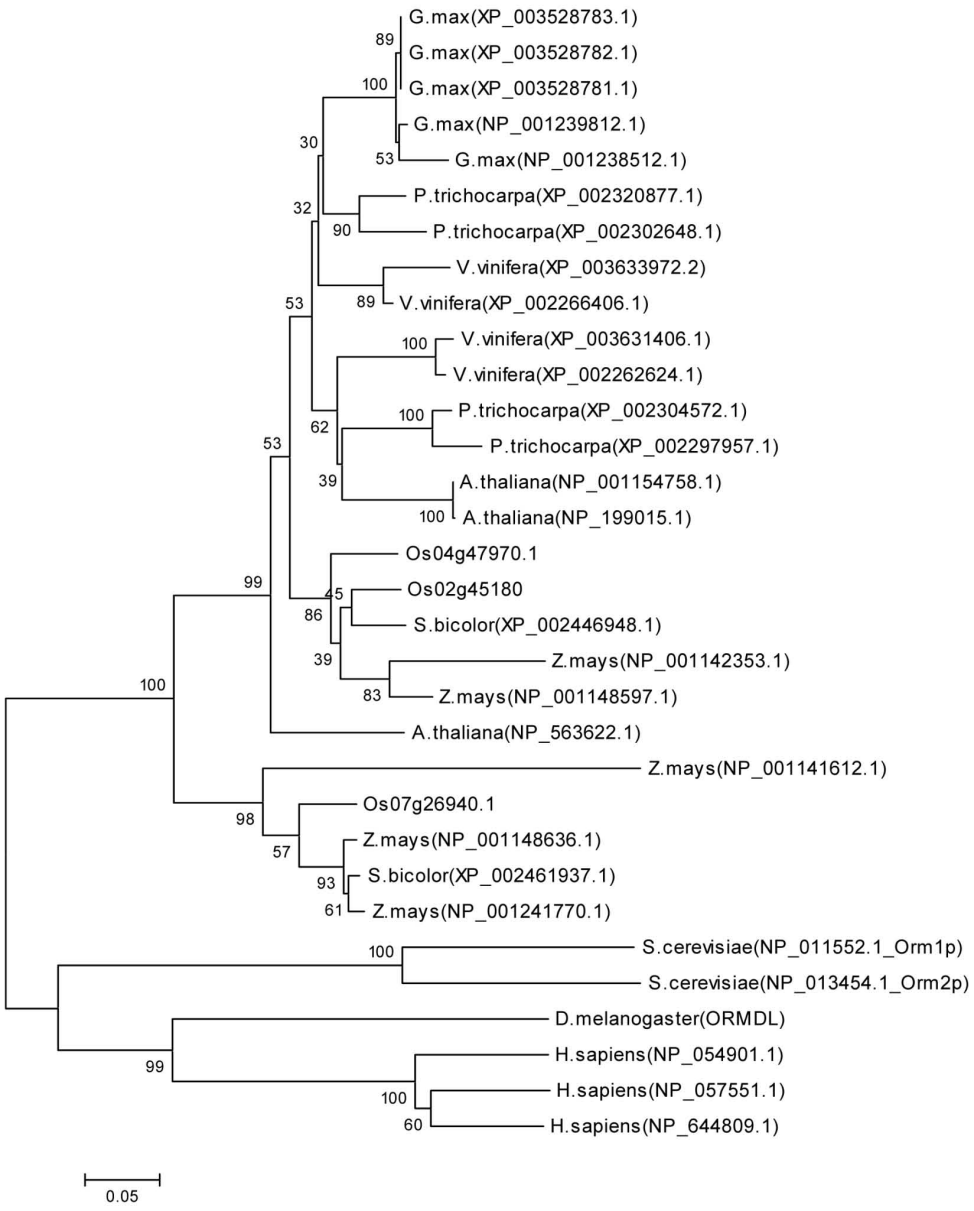
Phylogenetic relationship of rice ORMDL and other ORMDL from different species across kingdom. An un-rooted neighbour joining phylogenetic tree was constructed from the protein sequences of rice ORMDL obtained from GRAMENE rice database and ORMDL from other species selected from NCBI Reference Sequence database. Multiple sequence alignment was performed using the ClustalW in MEGA5 and the tree was generated using MEGA5. The numbers for interior branches indicate the bootstrap values (%) for 1000 replications.

*LOC_*Os07g26940 is a member of the ORMDL protein family, which is highly conserved in eukaryotes. Information from the GRAMENE rice database indicated that *ORMDL LOC_Os07g26940* has three splicing forms, and the results from sequence alignments of these transcripts indicated that the three transcripts of *ORMDL LOC_Os07g26940* shared the same sequence for about half of the gene starting from the beginning of the transcripts (Fig. S1). Previously, a BLAST search using the predicted amino acid sequences of *ORMDL LOC_Os07g26940* revealed two other sequences (encoded by *LOC_Os04g47970* and *LOC_Os02g45180)* with high percent sequence similarity to *ORMDL LOC_Os07g26940*, suggesting that at least three *ORMDL* genes are present in rice [27]. *ORMDL LOC_Os04g47970* has two splicing forms but *ORMDL LOC_Os02g45180* has only one. Similarly, results from comparing the two transcripts of *ORMDL LOC_Os04g47970* showed that they share about 75% of the sequences starting from the beginning of the transcripts (Fig. S2). The rice transcripts of *ORMDL* showed high homology in the coding sequences (Fig. S3). To understand the evolutionary relationships of this gene family in rice and other species across kingdoms, phylogenetic analysis was constructed, using deduced amino acid sequences from the longest transcript of each rice ORMDL. This analysis showed the phylogenetic relationship between rice ORMDL and other ORMDL from different species selected from NCBI Reference Sequence database. The results showed 4 main groups of genes from human, drosophila, yeast, and plants. As expected, rice ORMDL is closer to *Z. mays* and to *S. bicolor* than to *A. thaliana*. Interestingly, ORMDL LOC_Os07g26940 is in a separate clade from the other two rice ORMDL (Fig. 2).

### Expression analysis of rice *ORMDL* genes

**Figure 3:**
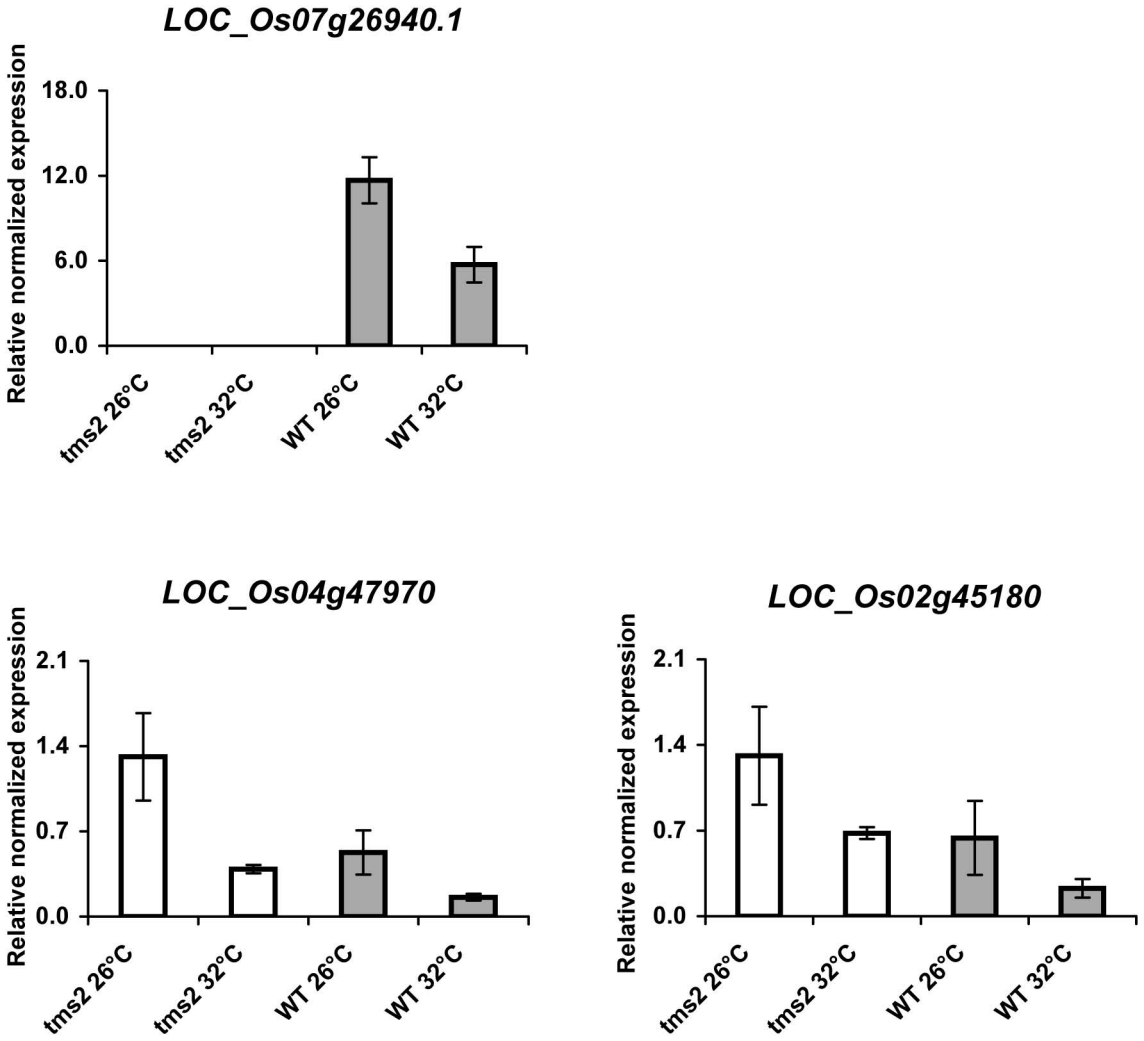
Expression of rice *ORMDL* genes in *tms2* and wild type rice plants. qRT-PCR analysis of rice *ORMDL* genes in anthers of *tms2* and wild type rice plants grown in control growth room at 26°C and 32°C. The expression level of *EF-1α* was used as internal control. All data are representative from at least two biological repeats, each based on three technical replicates; similar results were obtained in the repeated experiments. Bars indicate the standard error (n = 3), which was calculated from technical replicates. Each biological sample was a mixture of 3 plants.

To determine the patterns of expressions of rice *ORMDL* genes in *tms2* and wild type rice plants, expression of these genes by qRT-PCR was monitored in anthers of *tms2* and wild type plants grown in controlled growth room at 26 and 32°C. Using *EF-1α* as the reference gene, the results indicated that all the three *ORMDL* genes in rice showed similar patterns of expression by having higher expression at low temperature (26°C) than that at high temperature (32°C) conditions, and these genes showed higher expression in *tms2* than in wild type plants in both conditions. As expected, expression of *ORMDL LOC_Os 07g26940.1*, used as a representative splicing form of *ORMDL LOC_Os 07g26940*, was not detected in the *tms2* mutant rice plants (Fig. 3).

### RNAi transgenic rice plants

**Figure 4:**
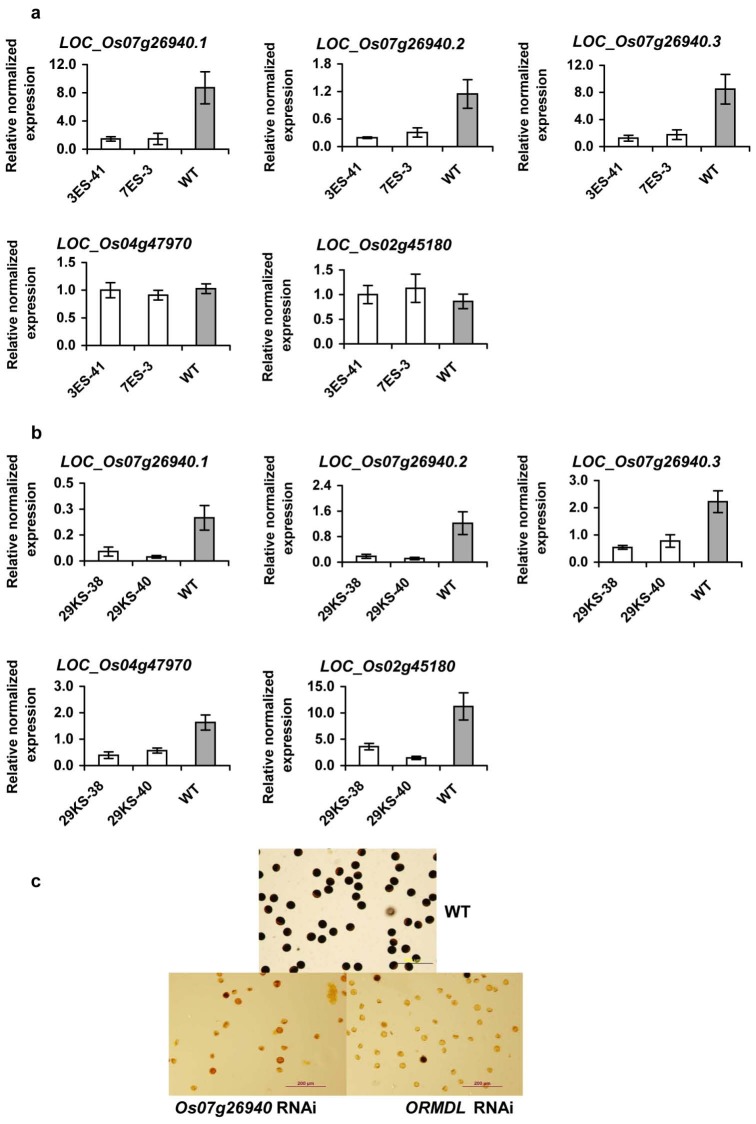
Expression analysis and pollen staining of RNAi transgenic rice plants. A) qRT-PCR analysis of *ORMDL LOC_Os07g26940* and other *ORMDL* gene expression in leaves of *LOC_Os07g26940* RNAi (3ES-41 and 7ES-3). B) qRT-PCR analysis of *ORMDL LOC_Os07g26940* and other *ORMDL* gene expression in leaves of *ORMDL* RNAi (29KS38 and 29KS-40) transgenic rice plants. 3ES-41 and 7ES-3 or 29KS38 and 29KS-40 are two independent transgenic lines from the same construct. The expression level of *EF-1α* was used as internal control. All data are representative from at least two biological repeats, each based on three technical replicates; similar results were obtained in the repeated experiments. Bars indicate the standard error (n = 3). which was calculated form technical replicates. Each biological sample was a mixture of 3 plants. C) Pollen staining of wild type, *LOC_Os07g26940* RNAi, and *ORMDL* RNAi transgenic rice plants. Similar results were obtained from the two tested transgenic lines form each construct. The pictures are a representative from each construct.

To investigate the role of *LOC_Os07g26930* and *ORMDL LOC_Os07g26940* on pollen development, RNAi technology was used to down-regulate *LOC_Os07g26930* or *ORMDL LOC_Os07g26940* expression in rice. For the gene-specific *ORMDL LOC_Os07g26940* RNAi construct, sequence present only in the transcripts of *ORMDL LOC_*Os07g26940, but not in *ORDML* genes (*LOC_Os04g47970* and *LOC_Os02g45180)* were used (Fig. S3). As this sequence is different from the other two rice *ORMDL* genes, we predicted that the RNAi only affects *ORMDL LOC_Os07g26940* expression. However, because three *ORMDL* genes are present in rice, knocking down only the *ORMDL LOC_Os07g26940* expression may not reveal all phenotypic effects. Thus, we generated an another RNAi construct using conserved sequences present in all rice *ORDML* genes (Fig. S3), to universally down-regulated all the *ORDML* gene transcripts. These RNAi sequences were under the control of ubiquitin promoter [28]. These RNAi constructs were transformed in to wild type (WT) *Nipponbare* plants. We obtained 27, 8 and 6 transgenic lines for *LOC_Os07g26930*-specificRNAi, *LOC_Os07g26940*-specificRNAi, and *ORMDL*-specific RNAi, respectively. In our experience, it was much more difficult to obtain *LOC_Os07g26940*-specificRNAi, and *ORMDL*-specific RNAi, transgenic lines than that from *LOC_Os07g26930*-specificRNAi construct. In natural condition, at flowering, none of transgenic plants for *LOC_Os07g26930*-specificRNAi were sterile. For, *LOC_Os07g26940*-specific RNAi, or *ORMDL*-specific RNAi, two independent transformed lines from each construct were selected for further studies on the basis of low levels of expression of the target genes and a single copy of the transgene construct. As expected, expressions of all three splicing forms of *ORMDL LOC_Os07g26940* were reduced in *LOC_Os07g26940*-specific RNAi transgenic plants, while expression of the other two ORMDL were not lower than that of wild type plants (Fig. 4A). For *ORMDL*-specific RNAi, expressions of all three *ORMDL* were reduced (Figure 4B). At flowering, the pollen of these plants was different from that of wild type rice plants in both morphology and staining. The wild type pollen was round, showing the presence of starch granules, while pollen of *LOC_Os07g26940*-specificRNAi, or *ORMDL*-specific RNAi, was abnormal showing small pear-shaped pollen grains lacking starch (Fig. 4C). In addition, these T0 transgenic plants were sterile when they were grown under glass house conditions. Thus, they were ratooned from one T0 plant to at least 6 plants from each of the original T0 transgenic plant. The plants were grown under glass house conditions for approximately one month. Subsequently, three plants from each line were moved to grow in control growth room at 26 and 32°C. At flowering, these plants were sterile, with no plants capable of setting seed. The panicle tissue of the T0 was not used for direct measurement as they were retained to observe seed setting and to allow for T1 seed production.

### Sphingolipid metabolism is perturbed in the *tms2* mutant and in RNAi rice lines

**Figure 5:**
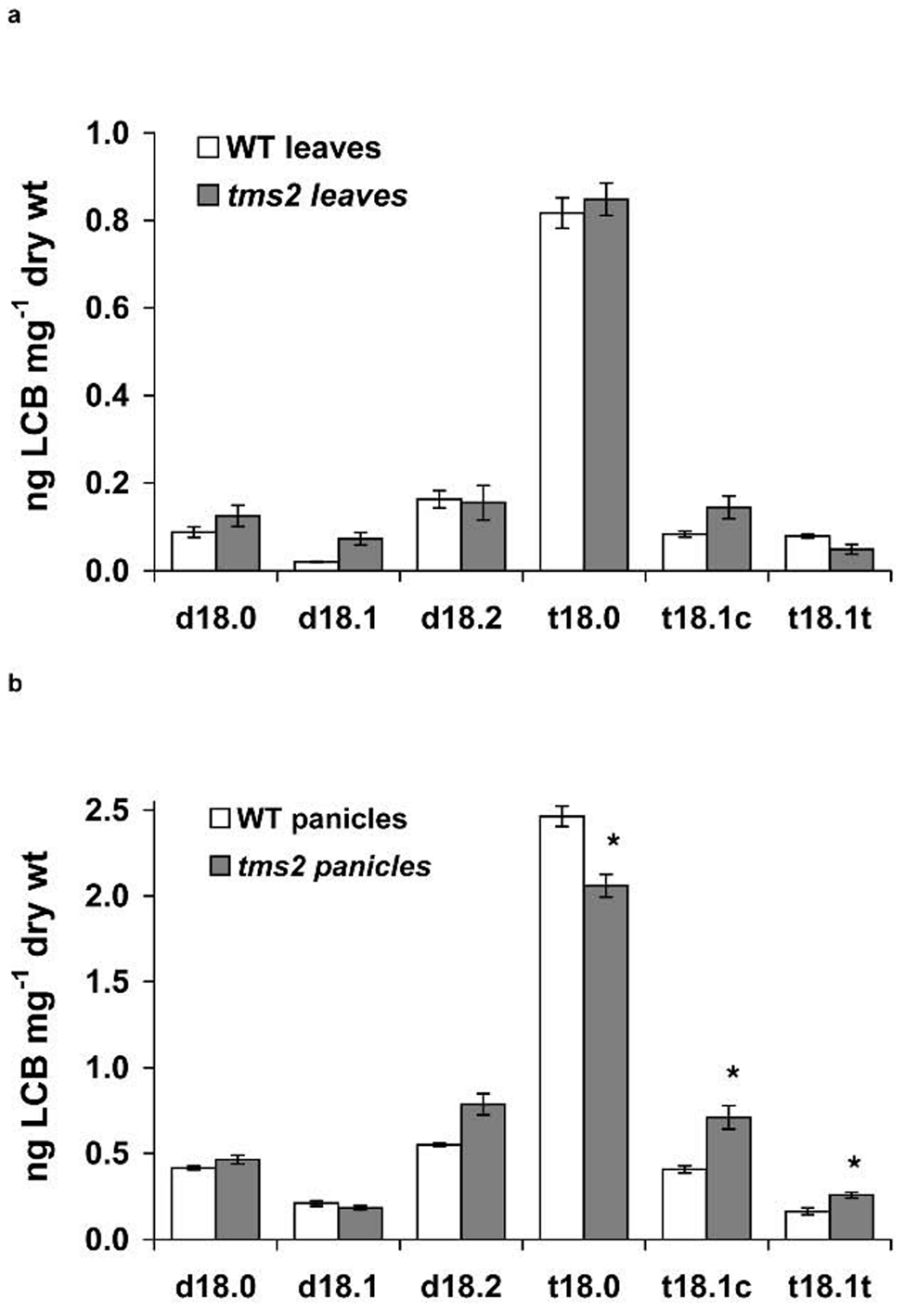
Sphingolipid long-chain base composition ng LCB mg^−1^ dry weight in wild type and *tms2* mutant rice plants in leaf tissue(A) and in panicles(B).

**Figure 6:**
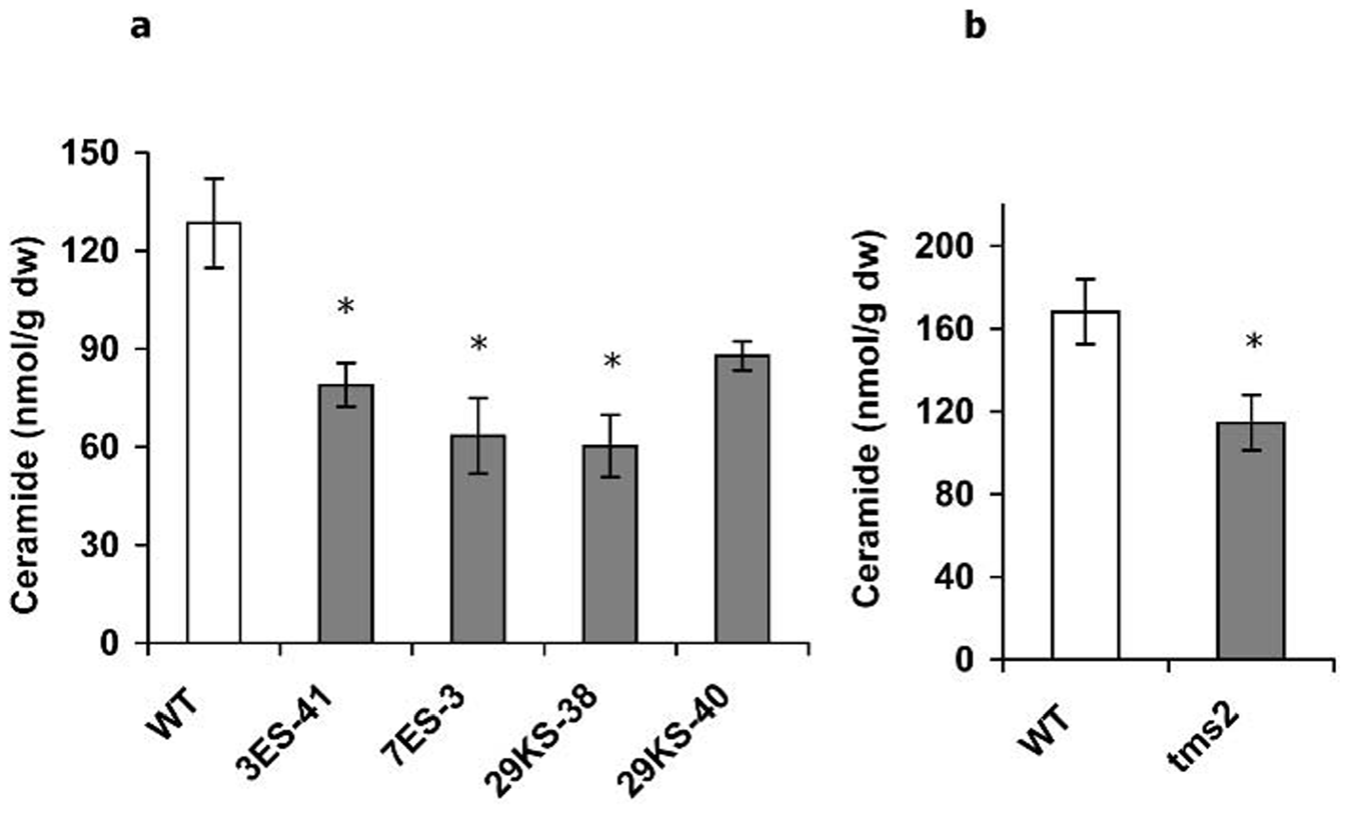
Total ceramide analysis. 6A) Total ceramide analysis in leafs of RNAi rice lines and WT rice plants. 6B) Total ceramide analysis in panicles of tms2 mutant and WT rice plants. Ceramide levels were determined from the leaves of *LOC_Os07g26940* RNAi (3ES-41 and 7ES-3) or *ORMDL* RNAi (29KS38 and 29KS-40) transgenic rice plants, and compared with equivalent tissue from WT. Results were expressed as absolute levels of ceramides (nmol/g dw). Similar analysis was performed on panicles of either WT or tms2 mutant rice, and data is presented as absolute levels of ceramides (nmol/g dw). Absolute levels (A, B) represent the mean of 6 technical replicates for each independent line. Bar indicates the standard error (n = 6). * indicates statistical significance at p-value of 0.05 as determined by t-test. 3ES-41 and 7ES-3 or 29KS38 and 29KS-40 are two independent transgenic lines from the same construct.

Recently, yeast ORM proteins were reported as negative regulators of sphingolipid synthesis [8], [9]. Since one *ORMDL* gene was deleted in the rice *tms2* mutant, one scenario is that this may affect or alter sphingolipid synthesis by releasing LCB synthesis from homeostatic regulation. To investigate whether the *tms2* mutant affects sphingolipid composition, total sphingolipid long-chain bases (LCBs) were analyzed from leafs and panicles of wild-type and *tms2* mutant rice plants. Sphingolipids comprise an LCB and an amide-linked a fatty acid moiety, usually also decorated with a sugar head group. The LCB component of sphingolipids is unique to these lipids, and its synthesis in yeast has been shown to be modulated by ORM1/2. Analysis of total LCBs showed that no significant difference in sphingolipid LCB composition was observed in leaves of *tms2* mutant and wild type rice plants (Fig. 5A). Importantly, panicles of *tms2* mutant rice plants had altered sphingolipid LCB composition, with higher levels of d18∶2, t18.1c, and t18∶1t but lower levels of t18∶0, compared with wild type rice plant(Fig. 5B). The other LCB species were similar in *tms2* mutant and wild type rice plants (Fig. 5B). In view of the alteration of LCB composition in the *tms2* mutant, and also previous studies in yeast which revealed altered ceramide levels in *orm1*Δ*/orm2* Δ mutants [8], [9], more targeted analysis was carried out, to measure the ceramide levels in this mutant and also RNAi-suppressed lines. The results showed that both RNAi-suppressed lines and *tms2* mutant plants showed a reduced accumulation of ceramides, relative to the WT (Fig. 6A–6B; note that in the case of the RNAi lines, analysis was carried out on leaf material). This was true for RNAi which suppressed all rice ORMDL transcripts, or specifically just the transcript derived from *LOC_Os07g26940* and absent in *tms2*. Breakdown of these data is shown in table 1 and 2. These data confirm the specific role for *LOC_Os07g26940* in modulating ceramide levels in rice.

**Table 1:** Ceramide composition in panicles of *Indica* WT and *tms*2 rice lines.

		c16:0	c18:0	c20:0	c20:1	c22:0	c22:1	c24:0	c24:1	c26:0	c26:1
**WT**	**d18:0**	0.41±0.09	0.01±0.00	0.13±0.01	0.14±0.02	0.04±0.01	0.02±0.01	0.15±0.01	0.02±0.00	0.23±0.02	0.02±0.01
	**d18:1**	0.13±0.03	0.03±0.02	0.28±0.09	8.86±1.69	0.23±0.07	1.01±0.10	0.60±0.24	0.60±0.13	0.19±0.10	0.10±0.03
	**d18:2**	1.61±0.50	0.38±0.03	4.29±0.31	77.85±4.52	0.69±0.07	13.29±1.03	0.52±0.08	11.19±1.37	0.21±0.07	2.25±0.47
	**t18:0**	0.62±0.06	0.40±0.08	2.52±0.28	0.13±0.02	5.29±0.92	0.46±0.08	18.46±8.21	0.88±0.16	5.95±2.77	1.39±0.29
	**t18:1**	0.13±0.01	0.07±0.04	0.10±0.01	0.72±0.08	0.15±0.03	1.56±0.11	0.94±0.19	2.23±0.22	0.51±0.11	0.30±0.05
***tms***	**d18:0**	0.40±0.19	0.01±0.00	0.07±0.01	0.07±0.03	0.02±0.00	0.01±0.00	0.09±0.02	0.02±0.01	0.17±0.04	0.01±0.00
	**d18:1**	0.07±0.02	0.01±0.00	0.13±0.03	4.39±1.61	0.16±0.04	0.54±0.13	0.47±0.17	0.35±0.10	0.19±0.10	0.06±0.10
	**d18:2**	1.59±0.59	0.24±0.06	2.85±0.58	54.81±10.10	0.44±0.09	8.46±1.45	0.35±0.06	7.26±1.33	0.09±0.02	1.48±0.29
	**t18:0**	0.49±0.10	0.30±0.09	1.31±0.33	0.11±0.03	3.36±0.81	0.25±0.04	13.78±6.70	0.55±0.18	4.46±2.39	0.64±0.13
	**t18:1**	0.13±0.03	0.02±0.00	0.05±0.01	0.45±0.10	0.13±0.04	0.87±0.20	0.61±0.13	1.67±0.46	0.33±0.10	0.28±0.07

Unit: nmol g dw^−1^, N: a minimum of 3± SE.

**Table 2:** Ceramide composition in leaves of *Japonica* WT and RNAi rice lines.

		c16:0	c18:0	c20:0	c20:1	c22:0	c22:1	c24:0	c24:1	c26:0	c26:1
WT	d18:0	0.02±0.00	0.00±0.00	0.02±0.00	0.02±0.00	0.02±0.00	0.02±0.01	0.20±0.06	0.00±0.00	0.20±0.05	0.01±0.00
	d18:1	0.04±0.00	2.02±1.02	0.02±0.00	1.88±0.15	0.15±0.02	0.51±0.07	0.98±0.27	0.22±0.02	0.14±0.01	0.07±0.00
	d18:2	0.01±0.01	0.14±0.20	2.13±0.79	55.09±12.86	0.54±0.20	14.40±3.31	0.17±0.05	5.05±1.16	0.02±0.00	0.61±0.14
	t18:0	0.21±0.07	0.11±0.02	0.50±0.08	0.05±2.86	4.76±1.13	0.27±0.68	28.87±7.07	0.91±0.09	4.09±1.01	0.22±0.03
	t18:1	0.02±0.00	0.01±0.00	0.05±0.01	0.48±0.53	0.06±0.24	1.41±0.21	0.36±1.50	1.04±0.22	0.08±0.21	0.11±0.02
3ES-41	d18:0	0.03±0.01	0.01±0.00	0.02±0.00	0.02±0.00	0.02±0.00	0.01±0.00	0.25±0.03	0.01±0.00	0.21±0.01	0.01±0.01
	d18:1	0.02±0.00	0.01±0.00	0.01±0.00	1.12±0.07	0.09±0.01	0.55±0.07	0.46±0.05	0.16±0.02	0.05±0.01	0.01±0.00
	d18:2	0.01±0.00	0.10±0.01	2.09±0.26	38.81±4.01	0.55±0.07	10.52±1.17	0.16±0.02	3.28±0.38	0.01±0.00	0.35±0.07
	t18:0	0.10±0.02	0.06±0.02	0.32±0.02	0.05±0.01	2.26±0.36	0.17±0.02	12.26±2.07	0.43±0.06	1.64±0.23	0.08±0.01
	t18:1	0.01±0.00	0.01±0.00	0.05±0.00	0.38±0.06	0.04±0.01	1.11±0.20	0.19±0.04	0.73±0.08	0.04±0.01	0.09±0.01
7ES-4	d18:0	0.03±0.00	0.02±0.01	0.02±0.01	0.03±0.01	0.03±0.00	0.03±0.02	0.45±0.07	0.02±0.00	0.41±0.11	0.02±0.00
	d18:1	0.04±0.01	0.02±0.00	0.07±0.01	0.69±0.17	0.12±0.04	0.28±0.07	0.61±0.19	0.24±0.05	0.11±0.04	0.03±0.01
	d18:2	0.03±0.00	0.08±0.01	0.57±0.23	21.46±8.24	0.17±0.07	7.44±2.99	0.03±0.01	3.31±1.16	0.05±0.01	0.30±0.09
	t18:0	0.25±0.05	0.09±0.05	0.47±0.13	0.18±0.03	3.55±0.75	0.53±0.11	14.25±4.58	1.12±0.00	2.27±0.00	0.23±0.00
	t18:1	0.04±0.01	0.04±0.01	0.21±0.11	0.37±0.18	0.08±0.04	1.37±0.43	0.38±0.09	0.99±0.49	0.07±0.02	0.14±0.05
29KS-38	d18:0	0.05±0.02	0.01±0.00	0.03±0.00	0.02±0.00	0.03±0.01	0.01±0.00	0.60±0.06	0.01±0.00	0.52±0.13	0.07±0.03
	d18:1	0.03±0.01	1.10±1.09	0.04±0.01	1.02±0.20	0.29±0.10	0.42±0.12	0.45±0.06	0.22±0.09	0.10±0.02	0.02±0.01
	d18:2	0.01±0.00	0.09±0.02	0.92±0.49	14.39±6.71	0.03±0.01	8.41±2.66	0.02±0.01	3.67±1.35	0.02±0.01	0.42±0.17
	t18:0	0.39±0.08	0.22±0.10	0.94±0.28	0.12±0.02	6.93±2.25	0.88±0.28	11.22±1.61	0.88±0.13	2.03±0.40	0.16±0.01
	t18:1	0.04±0.01	0.02±0.00	0.03±0.01	0.27±0.04	0.13±0.05	1.26±0.43	0.54±0.20	0.92±0.42	0.12±0.04	0.08±0.03
29KS-40	d18:0	0.03±0.01	0.00±0.00	0.02±0.00	0.03±0.01	0.04±0.00	0.01±0.00	0.31±0.01	0.01±0.00	0.27±0.01	0.01±0.00
	d18:1	0.02±0.00	0.73±0.72	0.04±0.01	1.74±0.05	0.12±0.02	0.54±0.03	0.31±0.05	0.12±0.00	0.05±0.01	0.01±0.00
	d18:2	0.01±0.00	0.14±0.00	0.13±0.00	46.73±0.00	0.03±0.00	12.50±0.00	0.01±0.00	4.02±0.00	0.01±0.00	0.45±0.00
	t18:0	0.13±0.01	0.10±0.05	0.50±0.02	0.05±0.00	3.02±0.09	0.18±0.00	10.70±3.06	0.33±0.07	1.25±0.18	0.09±0.02
	t18:1	0.01±0.00	0.02±0.00	0.02±0.01	0.48±0.04	0.03±0.00	1.30±0.08	0.21±0.01	0.90±0.01	0.05±0.00	0.10±0.00

Unit: nmol g dw^−1^, N: a minimum of 3± SE.

### Expression of genes in sphingolipid synthesis pathway

Rice *ORMDL* gene *LOC_Os07g26940* is proposed, on the basis of homology, to be a regulator of sphingolipid synthesis, and in line with this, deletion of this gene perturbed sphingolipid metabolism in the *tms2* mutant. Thus, we reasoned that the deletion might consequently also affect the expression of genes involved in sphingolipid biosynthesis. To determine the expression of genes involved in sphingolipid metabolism, we used genes involved in early steps of sphingolipid synthesis in Arabidopsis, which were homologous to those genes in *S. cerevisiae* [29], and then searched for homologs of these genes in rice using Arabidopsis and rice database (http://www.arabidopsis.org/; http://www.gramene.org/). A total of 12 rice genes encoded for four enzymes involved in early steps of sphingolipid synthesis were identified (Table 3) and tested for expression analysis in anthers.

**Table 3:** Identification of genes involved in early steps of sphingolipid biosynthesis in rice using homologous genes from *A. thaliana* and *S. cerevisiae*.

Gene	*S. cerevisiae*	*A. thaliana*	*O. sativa*
Serine palmitoyltransferase	LCB1	At4g36480	LOC_Os02g56300, LOC_Os10g11200, LOC_Os03g14800
	LCB2	At5g23670	LOC_Os11g31640, LOC_Os01g70380, LOC_Os01g70370
		At3g48780	LOC_Os11g31640, LOC_Os01g70380, LOC_Os01g70370
3-ketosphinganine reductase	TSC10	At3g06060	LOC_Os02g47350
		At5g19200	LOC_Os02g47350
Sphingolipid-C4-hydroxylase	SUR2	At1g14290	LOC_Os06g12250, LOC_Os02g51150
		At1g69640	LOC_Os06g12250, LOC_Os02g51150
Ceramide synthase	LAC1/LAG1	At3g25540	LOC_Os02g37080, LOC_Os02g49590
		At3g19260	LOC_Os03g15750
		At1g13580	-

**Figure 7:**
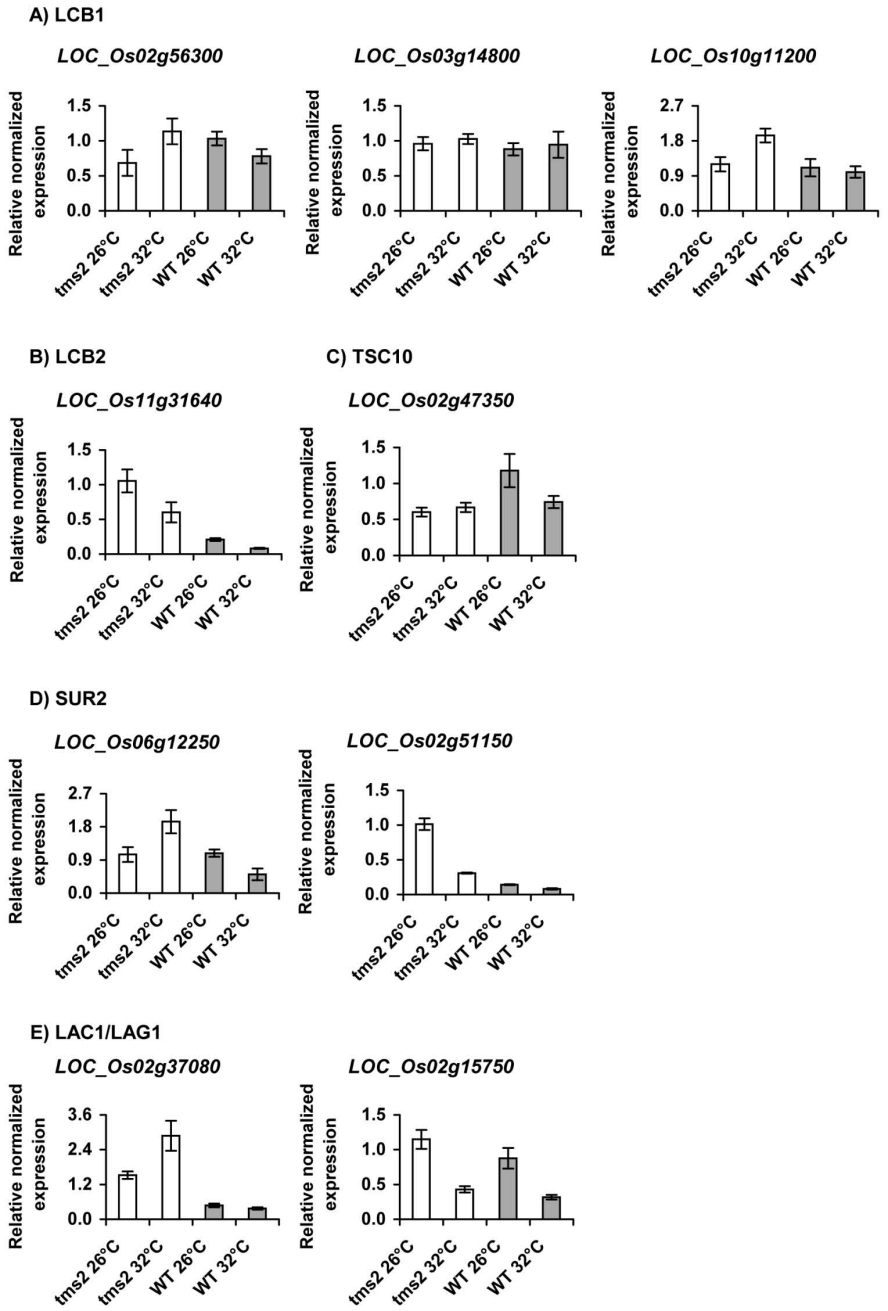
Expression analysis of genes involved in sphingolipid synthesis by quantitative RT-PCR. Expression of genes involved in early steps of sphingolipid synthesis including *LCB1*(A), *LCB2*(B), *TSC10* (C), *SUR2*(D), *LAC1/LAG1*(E) in anthers of *tms2* and wild type rice plants grown in control growth room at 26°C and 32°C. All data are representative from at least two biological repeats, each based on three technical replicates; similar results were obtained in the repeated experiments. Bars indicate the standard error (n = 3), which was calculated from technical replicates. Each biological sample was a mixture of 3 plants.

The expression of 9 genes encoding for four enzymes were detected (Fig. 7A–7E), while the primers specific for the other three genes including *serine palmitoyltransferase LOC_Os 01g70370, serine palmitoyltransferase LOC_Os 01g70380*, and *ceramide synthase LOC_Os 02g49590* were unable to provide good amplification. Two rice orthologs (*LOC_Os 02g56300* and *LOC_Os 10g11200*) of the *LCB1* subunit of serine palmitoyltransferase (SPT), showed higher expression at the non-permissive (i.e. sterile) temperature (32°C) in the *tms2* mutant compared with the permissive (fertile) temperature (26°C), and these *LCB1* transcripts were higher than those of wild type plants. Another rice ortholog of the *LCB1* (*serine palmitoyltransferase LOC_Os* 03g14800) showed similar expression levels in 26°C and 32°C both in wild type and mutant rice plants, and similar levels of expression in wild type and mutant rice plants (Fig. 7A). Interestingly, a rice ortholog (*serine palmitoyltransferase LOC_Os 11g31640*) of the *LCB2* showed higher expression at permissive (fertile) temperature (26°C) compared with the non-permissive (i.e. sterile) temperature (32°C) in both *tms2* mutant and in the wild type plants, and expressions of this gene were much higher in the mutant than those in the wild type plants at both conditions (Fig. 7B). A rice ortholog (*3-ketosphinganine reductase LOC_Os02g47350*) of *TSC10*, showed similar levels of expression at permissive (fertile) temperature (26°C) compared with the non-permissive (i.e. sterile) temperature (32°C) in *tms2* mutant, but this gene showed higher expression at permissive (fertile) temperature (26°C) compared with the non-permissive (i.e. sterile) temperature (32°C) in wild type plants (Fig. 7C). A rice ortholog (*sphingolipid-C4-hydroxylase LOC_Os06g12250*) of *SUR2* showed higher expression at the non-permissive (i.e. sterile) temperature (32°C) compared with the permissive (fertile) temperature (26°C) in the mutant, but in wild type, this gene showed higher expression at permissive (fertile) temperature (26°C) compared with the non-permissive (i.e. sterile) temperature (32°C). Surprisingly, the other tested rice ortholog (*sphingolipid-C4-hydroxylase LOC_Os02g51150*) of *SUR2* showed higher expression at permissive (fertile) temperature (26°C) compared with the non-permissive (i.e. sterile) temperature (32°C) in both *tms2* mutant and in the wild type plants but expression of this gene were much higher in the mutant than those in the wild type plants at both conditions (Fig. 7D). For *ceramide synthase*, a rice ortholog (*LOC_Os02g37080*) of LAC1/LAG1, showed higher expression at the non-permissive (i.e. sterile) temperature (32°C) in the *tms2* mutant compared with the permissive (fertile) temperature (26°C), and this expression was much higher than that of wild type plants. In wild type, this gene showed similar levels of expression, with very low level of expression at both conditions. The other tested rice ortholog (*ceramide synthase LOC_Os02g15750*) of LAC1/LAG1 showed higher expression at permissive (fertile) temperature (26°C) compared with the non-permissive (i.e. sterile) temperature (32°C) in both *tms2* mutant and in the wild type plants, and this gene showed similar levels of expressions in *tms2* mutant and in the wild type plants for each condition (Fig. 7E).

## Discussion

The temperature-sensitive genetic male sterility (TGMS) system provides a powerful tool for the production of hybrid rice, in turn leading to greater yields of this vital crop. However, the molecular mechanism(s) of TGMS are currently unknown, precluding the facile incorporation of this trait into germplasm. A previous study reported a TGMS phenotype of *Ugp1*-cosuppressed plants resulting from aberrant transcripts, which undergo temperature-sensitive splicing in florets [30]. A rice *ORMDL*, *LOC_Os07g26940* was predicted to have three splicing variants (http://www.gramene.org). Accordingly, we detected all the three transcripts by qRT-PCR. Interestingly, one splice variant showed strongly differential patterns of expression in anther at low and high temperatures, implying a role for *ORMDL LOC_Os07g26940* in response to temperatures in anther. Importantly, this gene showed high expression in panicle and anthers which, in addition to modulation by temperature, coincides with the TGMS phenotype of *tms2*.

The TGMS phenotype of *tms2* mutant rice plants was reported to be controlled by a recessive gene located in chromosome 7. Previously, the *tms2* mutant plants were reported to contain at least 70 kb deletion including seven genes annotated as expressed proteins [27]. However, only two of these genes (*LOC_Os07g26930 and ORMDL LOC_Os07g26940*) were strongly expressed in panicles and anthers and only the *ORMDL* gene *LOC_Os07g26940* showed differential expression in fertile and sterile conditions in anthers, suggesting a function in this tissue and in TGMS. Accordingly, knock down function of this gene or all *ORMDL* genes including *LOC_Os07g26940*, *LOC_Os04g47970*, and *LOC_Os02g45180*, by RNAi, affected pollen development resulting in male sterility. Reduction of only *ORMDL* gene expression in chromosome 7 (*LOC_Os07g26940*) or all *ORMDL* genes affected pollen development in both morphology and staining. Pollen grains of *LOC_Os07g26940* RNAi and *ORMDL* RNAi were different in sizes, shapes and staining, with none of these plants setting seeds. Interestingly, the male sterility of these RNAi plants was not modulated by temperature, with *LOC_Os07g26940* RNAi and *ORMDL* RNAi transgenic plants sterile at both low and high temperatures, suggesting that at least one other factor is needed for the conditional male sterility observed in the TGMS phenotype of *tms2*. Thus, deletion of *ORMDL LOC_Os07g26940* and this (currently unknown) factor are critical for the TGMS phenotype. Although genetic analysis indicates that the TGMS phenotype of *tms2* mutant plants was controlled by a single gene [31], [32], [27], it is possible that this phenotype is controlled by two closely linked genes not resolved by the current mapping population. Thus, a bigger population with greater recombination maybe required to further study this at the genetic level. Alternatively, the apparent inability of RNAi suppression of ORMDL to completely phenocopy the *tms2* TGMS may indicate subtle requirements for the total absence of this gene to deliver conditional fertility.

Recently, *ORMDL* was reported as a negative regulator of sphingolipid synthesis by inhibiting *serine palmitoyltransferase* (*LCB1/LCB2/TSC3*) [9]. Over expression of ORM1 and ORM2 in yeast reduced LCB and ceramide levels. Conversely, deletion of ORM1/2 elevated the levels of LCB and ceramides [9], although very similar studies have also reported decreased ceramides (in conjunction with elevated LCBs) in yeast mutants lacking ORMs [8]. Since a rice *ORMDL* gene is deleted in the *tms2* mutant, an alteration to sphingolipid homeostasis might be predicted. Accordingly, we found that sphingolipid synthesis was perturbed in the *tms2* mutant. Panicles of *tms2* mutant rice plants had higher d18∶2, t18.1c, and t18∶1t but lower t18∶0 than the wild type rice plants, although the overall level of LCBs was not significantly altered. Closer examination of the discrete ceramide species present in the panicles of WT and *tms2* mutant confirmed a concomitant decrease in ceramides levels, in agreement with total ceramide analysis, but also showed some discrete variation in the accumulation of individual ceramides. For example, the predominant C20∶1/d18∶2 ceramide species is reduced by ∼30% in the *tms2* mutant. This is particularly noteworthy, given the total LCB analysis of the *tms2* mutant revealed a moderate increase in this accumulation of this sphingoid base. However, since it is currently unclear if LCB modifying enzymes such as the sphingolipid D8-desaturase and the C4-hydroxylase utilise free LCBs and/or ceramides as substrates, it is not possible to make causal associations between these two different metabolic pools, or how these are impacted by the *tms2* mutation. It is interesting to note that the rice LAG1/LAC1 ceramide synthase ortholog *LOC_Os02g37080* showed upregulation in *tms2*, and although the substrate-specificity of this enzyme remains to be determined, it may play a role in the synthesis of a discrete subset of ceramides as seen in Arabidopsis [16]. More strikingly, and in clear agreement with the yeast studies of Han et al [8], we observed a significant reduction in the ceramide levels in rice plants with perturbation to the expression of the ORMDL gene *LOC_Os07g26940* – this was true for both the *tms2* mutant and also lines in which RNAi was used to suppress transcript accumulation. On the basis of these two independent interventions, we conclude that *LOC_Os07g26940* is specifically involved in the sphingolipid homeostasis, most likely in a manner analogous to that proposed by Han et al. [8].

In addition, the deletion in *tms2* mutant increased expression of *LCB1* in high temperature condition compared to that in wild type plants, and increased *LCB2* both in low and high temperature conditions in agreement with its previously established role in yeast [9]. Furthermore, the *tms2* deletion also affected expression of other genes involved in early steps of sphingolipid synthesis such as *SUR2* and *LAC1/LAG1*. Our results indicated that most of the genes involved in early steps of sphingolipid synthesis showed higher expression in the mutant than that in the wild type plants, particularly under high temperature treatment. In Arabidopsis, *LBC1* and *LCB2*, encoding subunits of serine palmitoyltransferase (SPT), were reported as important genes for male gametophyte development, involved in programmed cell death during pollen mitosis [23]. In addition, Arabidopsis *LCB2* null mutants resulted in gametophytic lethality by altering the endomembrane system of pollen and loss of pollen viability, which occurred early in pollen development during transition from the uni-nucleate microspore to the bicellular pollen grain [22]. Deletion of *ORMDL* gene in *tms2* mutant rice plants and RNAi suppression of *ORMDL* gene expression affected pollen development, resulting in sterility. The precise mechanism by which ORMDL *LOC_Os07g26940* modulates aspects of male fertility still remain to be resolved – based on our data, it appears that, like their yeast orthologs, higher plant ORMs are involved in sphingolipid homeostasis. However, the consequences of release from ORM regulation (reduced ceramides, and an altered LCB profile) is distinct from that observed in yeast, pointing the way for future studies. Given the significantly larger sphingolipidome of plant compared with *S. cerevisiae*, it is perhaps unsurprising that higher plants have evolved additional tiers of regulation for the control of these bioactive lipids.

In conclusion, our results indicate that *tms2* mutant plants lacking one member of the rice *ORMDL* gene family has altered sphingolipid composition. In addition, seven out of the nine expressed genes involved in sphingolipid synthesis pathway showed higher expression in the mutant compared to wild type plants, in agreement with the role of ORM as a homeostatic regulator of sphingolipid biosynthesis [9]. Our results indicate that *tms2* mutant plants lacking *ORMDL LOC_Os07g26940* affected the expression of several genes involved in early step of sphingolipid synthesis pathway, although the tested genes (orthologous for *LCB1, SUR2*, and *LAC1/LAG1*) had discrete expression patterns, suggesting an aspect of sub-functionalization most likely associated with plant-specific sphingolipid homeostasis. Our findings demonstrate an emerging role for *ORMDL* genes in plants as regulators of ceramide accumulation and also provide support for the important role of sphingolipids on pollen development.

## Materials and Methods

### Ethics Statement

N/A

### Plant materials and growth conditions

Wild type rice (*Oryza sativa* ssp *japonica* cv. Nipponbare, and *indica* ssp cv. Pathumthanee1) and *tms2* mutant (in *indica* background and backcrossed to Pathumthanee1, BC2F4) were planted in natural green house, and about one month before flowering some of these plants (at least 5 plants/lines/condition) were moved to controlled growth rooms at temperature 26°C or 32°C under 80% relative humidity, 12 h light/12 h darkness with a day until flowering.

### Expression analysis by quantitative RT-PCR

For expression of *LOC_Os07g26930* and *ORMDL LOC_Os07g26940*, specific primers for *LOC_Os07g26930* and each transcript of *ORMDL LOC_Os07g26940* were used for expression analysis in panicle and anther of wild type plants (Pathumthanee 1) grown in 24 and 32°C control growth rooms. Primers specific for *LOC_Os07g26930* and for each transcript of *ORMDL LOC_Os07g26940* were designed using Primer-BLAST (http://www.ncbi.nlm.nih.gov/tools/primer-blast/). In addition, primers specific for each transcript of *ORMDL LOC_Os07g26940* were used to test expression in other tissues of wild type plants such as root, stem, panicle, leaf, and anther. For expression of all the three *ORMDL* genes in *tms2* and wild type (Pathumthanee 1), primers specific for each *ORMDL* genes, were used and total RNAs were extracted from anther of *tms2* and wild type plants grown in 24 and 32°C control growth rooms. For expression of *ORMDL* genes in RNAi transgenic rice plants, total RNA were isolated from leaf, and qRT-PCR were performed using specific primers for each transcript of *Os07g26940* or primers specific for each of the other two *ORMDL* genes. For expression of genes involved in early steps of sphingolipid synthesis, primers specific for 12 rice genes identified as homologous genes of Arabidopsis, which are homologues to known genes involved in sphingolipid synthesis in *S. cerevisiae* were used for qRT-PCR using total RNAs extracted from anther of *tms2* and wild type plants (Pathumthanee 1) grown in 24 and 32°C control growth rooms. For all the data presented, one sample was a mixture of 3 plants for each genotype. Total RNAs were isolated using TRIZOL™ reagent (Invitrogen) following the manufacture's instructions. First-strand cDNA was synthesized using cDNA synthesis kit (Fermentas) and cDNAs transcribed from the total RNA (20 µl reaction volume). Gene specific primer were designed for amplicons about 200 bp. qRT-PCR experiments were performed on the BIO-RAD CFX 96 real time PCR systems, using SsoFast EvaGreen Supermix (Bio-Rad, Singapore). At the end of each experiment a melting curve was determined for each primer pair at a temperature stage from 60°C to 95°C to check the specificity of annealing. Primers targeting Actin1 (Os05g36290) were used to normalize the expression data of each gene in wild type plants. Because tms2 mutation affects expression of Actin1, thus as recommended [33] primers targeting Elongation Factor 1 α, EF-1α (Os03g08020) were used to normalize the expression data of genes compared expression in the mutant and wild type plants. For each gene, at least two biological replicates were performed, each with three technical replicated. Similar results were obtained in the repeated experiments. The average Ct values and standard deviation were calculated from three technical replicates. The quantification of gene expression was performed using the relative quantification method (2−ΔΔCT) [34]. All the tested primers were listed in Table S1.

### Phylogenetic Analysis

Phylogenetic Analysis was performed using protein sequences encoded by the longest splicing forms of each rice ORMDL obtained from GRAMENE rice database and ORMDL from other species selected from NCBI Reference Sequence database. The tree was generated by neighbour-joining (NJ) algorithm with p-distance method and pairwise deletion of gap, employing MEGA version 5 [35]. A bootstrap statistic analysis was performed with 1000 replicates to test the phylogeny.

### Generation of *Os07g26940*- RNAi and *ORMDL*-RNAi transgenic rice plants

To generate RNAi construct to suppress expression of *ORMDL LOC_Os07g26940*, all *ORMDL* genes, or *Os07g26930*, a 156-bp fragment specific only for *Os07g26940* cDNA was PCR amplified with primer *Os07g26940*-RNAi-F (5′-ggggtaccactagt CCACTCACCACGCGCCAC-3′, *Kpn*I, *Spe*I) and *Os07g26940*-RNAi-R (5′-ggggatccgagctc ACGTAGTAGGGGTAGGAC-3′, *Bam*HI, *Sac*I), a 250-bp fragment specific for all *ORMDL* cDNA was PCR amplified with primer *ORMDL*-RNAi-F(5′-ggggtaccactagtGTGAACAAGAACACGGAGT-3′, *Kpn*I, *Spe*I) and *ORMD* -RNAi-R (5′-ggggatccgagctcTGGTCATCAGCAGCAAAT -3′, *Bam*HI, *Sac*I), and a 422-bp fragment specific only for *Os07g26930* cDNA was PCR amplified with primer *Os07g26930*-RNAi-F(5′-ggggtaccactagtCCAAAAACCGCTCACACTCG-3′, *Kpn*I, *Spe*I), and *Os07g26930*-RNAi-R (5′- ggggatccgagctcGAATTCGATCCCAGGTGGCT -3′, *Bam*HI, *Sac*I), which harbors restriction sites (set in lowercase letters) for cloning. The resulting PCR products were first digested by *Spe*I and *Sac*I and ligated into vector pTCK303 [28] to get the transitional vector. Then, the PCR products were digested by *Kpn*I and *Bam*HI and ligated into the transitional vector. The resulting products were sent for sequencing to confirm sense and antisense orientations of the inserts in pTCK303. The constructs were transformed into rice plants (Nipponbare) using an *Agrobacterium*-mediated method.

### Pollen staining and microscopy

Mature pollens were obtained from anthers of 2–3 flowers. Anthers were disrupted on microscope slides using forceps and gently squashed in 4′, 6-diamidino-2-phenylindole (DAPI, Sigma-Aldrich) under a coverslip, and visualized with Olypus BX51 microscope.

### Analysis of sphingolipid long-chain bases

LCBs were liberated from 10mg of lyophilized leaves or panicle by alkaline hydrolysis, extraction method [36]. Briefly, this is preformed using 10% BaOH and dioxane 1∶1 v/v. The tubes are then capped and placed in a dry block at 110°C for 20 hours. TheLCBs are extracted in chloroform/dioxane/water (8/3/8, v/v/v). The LCBsare converted to dinitrophenyl derivatives with 0.2 ml of 0.5% (v/v) methanolic 1-fluoro-2, 4-dinitrobenzene and 0.8 ml of 2 M boric acid/KOH, at 60°C for 30 mins. LCBs are then extracted by phase partitioning with CHCl3/methanol/H2O, 2∶1∶1 (v/v/v). The organic phase is removed and washed with an equal volume of 0.1 M KOH and 0.5 M KCl. The organic phase is then blown down and resuspended with methanol, and analyzed by reversed-phase HPLC. Separation was performed using a C18 RP 250×4-mm column with a flow rate of 1 ml/min and a concave gradient from 80 to 100% methanol/acetonitrile/2-propanol, 10∶3∶1 (v/v/v), against water in 45 min. The elution was monitored with an Agilent 1200 DAD measuring at 350 nm and by ESI-MS/MS MRM on an ABSciex 4000 QTRAP. Three replicate extractions and LCB analysis were performed of each extraction method. Data presented as average (+/− standard error) in ng LCB per mg of dry weight.

### Analysis of ceramide

Samples extracted were ground in liquid N2 to a fine powder. 10 mg of each sample was used for each replicate analysis. Analysis of the ceramide fraction was performed using LC-MS/MS as previously described [37]. Briefly, the ceramides were extracted in the lower phase of isopropanol/hexane/water (55∶20∶25 v/v/v) at 60 degrees C. Samples were dried down under a stream of nitrogen. The crude extract was de-esterified in 2 ml of 33% methylamine solution in ethanol/water (7∶3 v/v) at 50 degrees for 1 hour. After hydrolysis the samples were dried down under a stream of nitrogen. The ceramides were resuspended in 1 mL of tetrahydrofuran (THF)/methanol/water (2∶1∶2 v/v/v) and 0.1% formic acid and 50 µL was analyzed on a 4000 QTRAP LC-MS/MS system (ABSciex) after HPLC using an Agilent 1200 fitted with a 100- µL sample loop. Separation was achieved on a SUPELCOSIL ABZ+Plus column 150×3 mm, 5- µm particle size, The sample was eluted at 1 mL min−1 with a binary gradient system consisting of solvent A, THF/methanol/5 mM ammonium formate (3∶2∶5v/v/v), and 0.1% formic acid, and solvent B, THF/methanol/5 mM ammonium formate (7∶2∶1v/v/v), and 0.1% formic acid. held at 40°C. The sample was eluted at 1 mL min^−1^ with a binary gradient system consisting of solvent A, THF/methanol/5 mM ammonium formate (3∶2∶5v/v/v), and 0.1% formic acid, and solvent B, THF/methanol/5 mM ammonium formate (7∶2∶1v/v/v), and 0.1% formic acid. The gradient started at 40% B rising to 75% B over 10 min. At the end of the gradient, the %B was increased to 100% over 1 min and held at 100% B for an additional 1 min to ensure complete elution of all compounds from the column. The probe was vertically positioned 11 mm from the orifice and charged with 5000 V. The temperature was held at 650°C, GS1 was set at 90 p.s.i., GS2 at 50 p.s.i., curtain gas at 20 p.s.i., and the interface heater was engaged. A minimum of 3 replicate extractions and analysis were performed. Data presented as average (+/− standard deviation). A t-test was performed to determine statistical significance, and in all cases data presented has a p-value of 0.05.

## Supporting Information

Figure S1 Sequence alignment of the three transcripts of Os07g26940. Sequences of rice ORMDL were obtained from GRAMENE rice database.
(DOC)

Figure S2 Sequence alignment of the two transcripts of Os04g47970. The sequences were obtained from GRAMENE rice database.
(DOC)

Figure S3 Sequence alignment of the representative three transcripts of *ORMDL* genes. Highlight in yellow indicates sequences specific for Os07g26940. Highlight in gray indicates sequences used to make RNAi to knockdown all ORMDL genes. Green and red indicates primer sequences used for Os07g26940RNAi and ORMDL RNAi, respectively. The sequences were obtained from GRAMENE rice database.
(DOC)

Table S1 qRT-PCR primer sequence.
(DOC)

## References

[1] Galanter J, Choudhry S, Eng C, Nazario S, Rodríguez-Santana JR, et al (2008) ORMDL3 gene is associated with asthma in three ethnically diverse populations. Am J Respir Crit Care Med 177: 1194–200. 18310477 10.1164/rccm.200711-1644OC PMC2408437

[2] Wu H, Romieu I, Sienra-Monge JJ, Li H, del Rio-Navarro BE, et al (2009) Genetic variation in ORM1-like 3 (ORMDL3) and gasdermin-like (GSDML) and childhood asthma. Allergy 64: 629–35. 19133921 10.1111/j.1398-9995.2008.01912.x PMC2697826

[3] Fang Q, Zhao H, Wang A, Gong Y, Liu Q (2011) Association of genetic variants in chromosome 17q21 and adult-onset asthma in a Chinese Han population. BMC Med Genet 12(1): 133. 21985515 10.1186/1471-2350-12-133 PMC3207945

[4] Moffatt MF, Kabesch M, Liang L, Dixon AL, Strachan D, et al (2007) Genetic variants regulating ORMDL3 expression contribute to the risk of childhood asthma. Nature 448: 470–473. 17611496 10.1038/nature06014

[5] Verlaan DJ, Berlivet S, Hunninghake GM, Madore AM, Larivière M, et al (2009) Allele-specific chromatin remodeling in the ZPBP2/GSDMB/ORMDL3 locus associated with the risk of asthma and autoimmune disease. Am J Hum Genet 85: 377–93. 19732864 10.1016/j.ajhg.2009.08.007 PMC2771592

[6] Cantero-Recasens G, Fandos C, Rubio-Moscardo F, Valverde MA, Vicente R (2010) The asthma-associated ORMDL3 gene product regulates endoplasmic reticulum-mediated calcium signaling and cellular stress. Hum Mol Genet 19: 111–121. 19819884 10.1093/hmg/ddp471

[7] Hjelmqvist L, Tuson M, Marfany G, Herrero E, Balcells S, et al (2002) ORMDL proteins are a conserved new family of endoplasmic reticulum membrane proteins. Genome Biol 3: RESEARCH0027. 12093374 10.1186/gb-2002-3-6-research0027 PMC116724

[8] Han S, Lone MA, Schneiter R, Chang A (2010) Orm1 and Orm2 are conserved endoplasmic reticulum membrane proteins regulating lipid homeostasis and protein quality control. Proc Natl Acad Sci U S A 107: 5851–6. 20212121 10.1073/pnas.0911617107 PMC2851911

[9] Breslow DK, Collins SR, Bodenmiller B, Aebersold R, Simons K, et al (2010) Orm family proteins mediate sphingolipid homeostasis. Nature 463: 1048–53. 20182505 10.1038/nature08787 PMC2877384

[10] Liu M, Huang C, Polu SR, Schneiter R, Chang A (2012) Regulation of sphingolipid synthesis via Orm1 and Orm2 in yeast. J Cell Sci 125: 2428–2435. 22328531 10.1242/jcs.100578 PMC3383258

[11] Spassieva SD, Hille J (2003) Plant sphingolipids today—Are they still enigmatic? Plant Biol 5: 125–136.

[12] Sperling P, Heinz E (2003) Plant sphingolipids: structural diversity, biosynthesis, first genes and functions. Biochim Biophys Acta 1632: 1–15. 12782146 10.1016/s1388-1981(03)00033-7

[13] Lynch DV, Dunn TM (2004) An introduction to plant sphingolipids and a review of recent advances in understanding their metabolism and function. New Phytol 161: 677–702. 10.1111/j.1469-8137.2004.00992.x 33873728

[14] Shi L, Bielawski J, Mu J, Dong H, Teng C, et al (2007) Involvement of sphingoid bases in mediating reactive oxygen intermediate production and programmed cell death in Arabidopsis. Cell Res 17: 1030–1040. 18059378 10.1038/cr.2007.100

[15] Aubert A, Marion J, Boulogne C, Bourge M, Abreu S, et al (2011) Sphingolipids involvement in plant endomembrane differentiation: the BY2 case. Plant J 65: 958–71. 21205030 10.1111/j.1365-313X.2011.04481.x

[16] Markham JE, Molino D, Gissot L, Bellec Y, Hématy K, et al (2011) Sphingolipids containing very-long-chain Fatty acids define a secretory pathway for specific polar plasma membrane protein targeting in Arabidopsis. Plant Cell 23: 2362–78. 21666002 10.1105/tpc.110.080473 PMC3160045

[17] Chen M, Markham JE, Cahoon EB (2012) Sphingolipid Δ8 unsaturation is important for glucosylceramide biosynthesis and low-temperature performance in Arabidopsis. Plant J 69: 769–81. 22023480 10.1111/j.1365-313X.2011.04829.x

[18] Guo L, Mishra G, Markham JE, Li M, Tawfall A, et al (2012) Connections between sphingosine kinase and phospholipase D in the abscisic acid signaling pathway in Arabidopsis. J Biol Chem 287: 8286–96. 22275366 10.1074/jbc.M111.274274 PMC3318714

[19] Coursol S, Fan LM, Le Stunff H, Spiegel S, Gilroy S, et al (2003) Sphingolipid signalling in Arabidopsis guard cells involves heterotrimeric G proteins. Nature 423(6940): 651–4. 12789341 10.1038/nature01643

[20] Chen M, Markham JE, Dietrich CR, Jaworski JG, Cahoon EB (2008) Sphingolipid long-chain base hydroxylation is important for growth and regulation of sphingolipid content and composition in Arabidopsis. Plant Cell 20: 1862–78. 18612100 10.1105/tpc.107.057851 PMC2518246

[21] Chao DY, Gable K, Chen M, Baxter I, Dietrich CR, et al (2011) Sphingolipids in the root play an important role in regulating the leaf ionome in Arabidopsis thaliana. Plant Cell 23: 1061–81. 21421810 10.1105/tpc.110.079095 PMC3082254

[22] Dietrich C, Han G, Chen M, Berg HR, Dunn TM, et al (2008) Loss-of-function mutations and inducible RNAi suppression of Arabidopsis LCB2 genes reveal the critical role of sphingolipids in gametophytic and sporophytic cell viability. Plant J 54: 284–298. 18208516 10.1111/j.1365-313X.2008.03420.x

[23] Teng C, Dong H, Shi L, Deng Y, Mu J, et al (2008) Serine palmitoyltransferase, a key enzyme for de novo synthesis of sphingolipids, is essential for male gametophyte development in Arabidopsis. Plant Physiol 146: 1322–1332. 18218968 10.1104/pp.107.113506 PMC2259075

[24] Wang XL, Li XB (2009) The GhACS1 gene encodes an acyl-CoA synthetase which is essential for normal microsporogenesis in early anther development of cotton. Plant J 57: 473–86. 18826432 10.1111/j.1365-313X.2008.03700.x

[25] Imamura T, Kusano H, Kajigaya Y, Ichikawa M, Shimada H (2007) A rice dihydrosphingosine c4 hydroxylase (dsh1) gene, which is abundantly expressed in the stigmas, vascular cells and apical meristem may be involved in fertility. Plant Cell Physiol 48: 1108–1120. 17609219 10.1093/pcp/pcm084

[26] Virmani SS, Kumar I (2004) Development and use of hybrid rice technology to increase rice productivity in the tropics. International Rice Research Notes 29: 10–19.

[27] Pitnjam K, Chakhonkaen S, Toojinda T, Muangprom A (2008) Identification of a deletion in tms2 and development of gene-based markers for selection. Planta 228: 813–822. 18642025 10.1007/s00425-008-0784-3

[28] Wang Z, Xu Y, Jiang R, Xu Z, Chong K (2004) A practical vector for efficient knockdown of gene expression in rice (Oryza sativa L.). Plant Molecular Biology Reports 22: 409–417.

[29] Zauner S, Ternes P, Warnecke D (2010) Biosynthesis of sphingolipids in plants (and some of their functions). Adv Exp Med Biol 688: 249–263. 20919660 10.1007/978-1-4419-6741-1_18

[30] Chen R, Zhao X, Shao Z, Wei Z, Wang Y, et al (2007) Rice UDP-glucose pyrophosphorylase1 is essential for pollen callose deposition and its cosuppression results in a new type of thermosensitive genic male sterility. Plant Cell 19: 847–61. 17400897 10.1105/tpc.106.044123 PMC1867369

[31] Yamaguchi Y, Ikeda R, Hirasawa H, Minami M, Ujihara A (1997) Linkage analysis of thermosensitive genic male sterility gene, *tms-2* in rice(*Oryza sativa* L.). Breeding Sci 47: 371–373.

[32] Lopez MT, Toojinda T, Vanavichit A, Tragoonrung S (2003) Microsatellite markers flanking the *tms2* gene facilitated tropical TGMS rice line development. Crop Sci 43: 2267–2271.

[33] Caldana C, Scheible WR, Mueller-Roeber B, Ruzicic S (2007) A quantitative RT-PCR platform for high-throughput expression profiling of 2500 rice transcription factors. Plant Methods 3(1): 7 10.1186/1746-4811-3-7 17559651 PMC1914063

[34] Livak KJ, Schmittgen TD (2001) Analysis of relative gene expression data using real-time quantitative PCR and the 2(-Delta Delta C(T)) Method. Methods 25: 402–408 10.1006/meth.2001.1262 11846609

[35] Tamura K, Peterson D, Peterson N, Stecher G, Nei M, et al (2011) MEGA5: molecular evolutionary genetics analysis using maximum likelihood, evolutionary distance, and maximum parsimony methods. Mol Biol Evol 28: 2731–2739 10.1093/molbev/msr121 21546353 PMC3203626

[36] Ternes P, Sperling P, Albrecht S, Franke S, Cregg JM, et al. (2006) Identification of fungal sphingolipid C9-methyltransferases by phylogenetic profiling. J Biol Chem 281: 5582–5592. 10.1074/jbc.M512864200 16339149

[37] Markham JE, Jaworski JG (2007) Rapid measurement of sphingolipids from Arabidopsis thaliana by reversed-phase high-performance liquid chromatography coupled to electrospray ionization tandem mass spectrometry. Rapid Commun. Mass Spectrom 21: 1304–1314. 17340572 10.1002/rcm.2962

