# Supplementary material for: Rice ORMDL Controls Sphingolipid Homeostasis Affecting Fertility Resulting from Abnormal Pollen Development
Source: PLoS One. 2014 Sep 5;9(9):e106386. doi: 10.1371/journal.pone.0106386 (PMC4156325; doi:10.1371/journal.pone.0106386)
Supplement: Figure S1 — Sequence alignment of the three transcripts of Os07g26940. Sequences of rice ORMDL were obtained from GRAMENE rice database. (DOC) [file pone.0106386.s001.doc]

t26940.3 GTACACAATAGGGTATCATCATCACACCATCACGAGCACCACCACCACTCACCACGCGCC

t26940.1 GTACACAATAGGGTATCATCATCACACCATCACGAGCACCACCACCACTCACCACGCGCC

t26940.2 GTACACAATAGGGTATCATCATCACACCATCACGAGCACCACCACCACTCACCACGCGCC

************************************************************

t26940.3 ACCCACCCCCGCACCGCACGCCAAGATCCGACCGCCGCCGCCGCCGCCGCCGGAGTAGGG

t26940.1 ACCCACCCCCGCACCGCACGCCAAGATCCGACCGCCGCCGCCGCCGCCGCCGGAGTAGGG

t26940.2 ACCCACCCCCGCACCGCACGCCAAGATCCGACCGCCGCCGCCGCCGCCGCCGGAGTAGGG

************************************************************

t26940.3 AGAGGGAGAGGAGATGGGGCGGAGGGCGGGGTCCTACCCCTACTACGTGGAGGCGGCGCC

t26940.1 AGAGGGAGAGGAGATGGGGCGGAGGGCGGGGTCCTACCCCTACTACGTGGAGGCGGCGCC

t26940.2 AGAGGGAGAGGAGATGGGGCGGAGGGCGGGGTCCTACCCCTACTACGTGGAGGCGGCGCC

************************************************************

t26940.3 GCCGGTGGACGTGAACAAGAACACGGAGTGGTTCATGTACCCCGGGGTGTGGACCACCTA

t26940.1 GCCGGTGGACGTGAACAAGAACACGGAGTGGTTCATGTACCCCGGGGTGTGGACCACCTA

t26940.2 GCCGGTGGACGTGAACAAGAACACGGAGTGGTTCATGTACCCCGGGGTGTGGACCACCTA

************************************************************

t26940.3 CATCCTCCTCCTCTTCTTCGCCTGGCTGCTCGTCCTCTCCGTCTCCGCCTGCTCCCCCGG

t26940.1 CATCCTCCTCCTCTTCTTCGCCTGGCTGCTCGTCCTCTCCGTCTCCGCCTGCTCCCCCGG

t26940.2 CATCCTCCTCCTCTTCTTCGCCTGGCTGCTCGTCCTCTCCGTCTCCGCCTGCTCCCCCGG

************************************************************

t26940.3 GATCGCGTGGACCGTCGTCAACCTCGCCCACTTCGCC-----------------------

t26940.1 GATCGCGTGGACCGTCGTCAACCTCGCCCACTTCGCCATCACTTATCACTTCTTCCATTG

t26940.2 GATCGCGTGGACCGTCGTCAACCTCGCCCACTTCGCC-----------------------

*************************************

t26940.3 ------------------------------------------------------------

t26940.1 GAAGAAGGGAACTCCATTTGCTGCTGATGACCAAGGCATCTACAACAGACTAACTTGGTG

t26940.2 ------------------------------------------------------------

t26940.3 ------------------------------------------------------------

t26940.1 GGAACAAATTGATAACGGGCAGCAACTTACTCGTAATAGGAAGTTCTTGACTGTGGTACC

t26940.2 ------------------------------------------------------------

t26940.3 ---------GTACCTGATCGCGTCACACTTGACCGACTACAAACAGCCAATGCT-TTTCC

t26940.1 TGTGGTGCTGTACCTGATCGCGTCACACTTGACCGACTACAAACAGCCAATGCT-TTTCC

t26940.2 ----------------ATCACTTATCACTTCTTCCATTGGAAGAAGGGAACTCCATTTGC

*** * * ***** * * * ** ** ** * *** *

t26940.3 TCAACACCATTGCAGTTCTGGTACTAGTGGTAGCAAAGCTGCCAAACATGCACAAGGTCC

t26940.1 TCAACACCATTGCAGTTCTGGTACTAGTGGTAGCAAAGCTGCCAAACATGCACAAGGTCC

t26940.2 TGCTGATGACCAAGGCATCTACAACAG-ACTAACTTGGTGGGAACAAATTGATAACGGGC

* * * * * ** ** * * * * * ** * ** * *

t26940.3 GTATATTTGGAATCAATGCAGATATCTGAGGACCATATGAAGAACATCAGGGCACAAGCT

t26940.1 GTATATTTGGAATCAATGCAGATATCTGAGGACCATATGAAGAACATCAGGGCACAAGCT

t26940.2 AGCAACTT--ACTCGTAATAGGAAGTTCTTGAC--TGTGGTACCTGTGGTGCTGTAAGTT

* ** * ** ** * * *** * ** * * *** *

t26940.3 TCTGTGTGTTTGCAACGAGGGACAAGCGAACTTTGTGCAAACACGGTATCTAAAGTAGTG

t26940.1 TCTGTGTGTTTGCAACGAGGGACAAGCGAACTTTGTGCAAACACGGTATCTAAAGTAGTG

t26940.2 CCATTTCCATTTCAAC------CTAATGTACTTCATCATGGGATTTTGTTGTAGTTGTAG

* * ** **** * * * **** * * * * * * *

t26940.3 AAAAAACTAAACTTTTTCACCACTGCAATCTGTAGTATGTGCATAGGTGTAGGAAGAACC

t26940.1 AAAAAACTAAACTTTTTCACCACTGCAATCTGTAGTATGTGCATAGGTGTAGGAAGAACC

t26940.2 AAAGAACTCAAAATGT--ATAATTGTTGT-TACAATAAGTATTTCTGGGGTGGAAGCATC

*** **** ** * * * * ** * * * ** ** * * * ***** * *

t26940.3 TGTAAATGTTACTGTAGCTTATAATTTGCCTACAGGTTGGAGGCTGCTGCATGTTTGCTC

t26940.1 TGTAAATGTTACTGTAGCTTATAATTTGCCTACAGGTTGGAGGCTGCTGCATGTTTGCTC

t26940.2 -GTCCATTGTGAAGGAATTTATTT------------------------------------

** ** * * * ****

t26940.3 ATGCCACTTCCTTTAAGAACTCTAGTGGAGAGTGTAGACTGTAACTTAAATAAACATGTA

t26940.1 ATGCCACTTCCTTTAAGAACTCTAGTGGAGAGTGTAGACTGTAACTTAAATAAACATGTA

t26940.2 ------------------------------------------------------------

t26940.3 ATCTTGTGGATATGGAAAGTTTGGTGGTTGTTAAAATGGTGAACCGGAGTTCAGTTCCTG

t26940.1 ATCTTGTGGATATGGAAAGTTTGGTGGTTGTTAAAATGGTGAACCGGAGTTCAGTTCCTG

t26940.2 ------------------------------------------------------------

t26940.3 CAA

t26940.1 CAA

t26940.2 ---

**Figure S1**. Sequence alignment of the three transcripts of Os07g26940.

Sequences of rice ORMDL were obtained from GRAMENE rice database.
