# Supplementary material for: Rice ORMDL Controls Sphingolipid Homeostasis Affecting Fertility Resulting from Abnormal Pollen Development
Source: PLoS One. 2014 Sep 5;9(9):e106386. doi: 10.1371/journal.pone.0106386 (PMC4156325; doi:10.1371/journal.pone.0106386)
Supplement: Figure S2 — Sequence alignment of the two transcripts of Os04g47970. The sequences were obtained from GRAMENE rice database. (DOC) [file pone.0106386.s002.doc]

T4g47970.2 GCGCCGTGCGGTGTGACGGCATTGGTGGCGCGGTGTGCGTCGCGGCACAC

T4g47970.1 GCGCCGTGCGGTGTGACGGCATTGGTGGCGCGGTGTGCGTCGCGGCACAC

**************************************************

T 4g47970.2 AGCCGCACAGGCGCACAGCCAGGCAGCCAGCCCGTCGGTCTCCTCTCCTC

T 4g47970.1 AGCCGCACAGGCGCACAGCCAGGCAGCCAGCCCGTCGGTCTCCTCTCCTC

**************************************************

T4g47970.2 TCCCCCTCGCGTTTTTTCTTTTCGCCTCCATCTTTGAACCTTCTCCTCGC

T4g47970.1 TCCCCCTCGCGTTTTTTCTTTTCGCCTCCATCTTTGAACCTTCTCCTCGC

**************************************************

T4g47970.2 CTTCTTCTTCCTCCTCCTCCCTCCGCTCCGCTTCTCTCTGACCAATAATC

T4g47970.1 CTTCTTCTTCCTCCTCCTCCCTCCGCTCCGCTTCTCTCTGACCAATAATC

**************************************************

T4g47970.2 TGGGCAAACCCCTCTCTCGCGCCTTCTCCCTCCCACCTCTATAAATCCCC

T4g47970.1 TGGGCAAACCCCTCTCTCGCGCCTTCTCCCTCCCACCTCTATAAATCCCC

**************************************************

T4g47970.2 CTCCATTCCAATCCCACGGGCTCAGGCCGCGCCACCAGCAGCAGCACACA

T4g47970.1 CTCCATTCCAATCCCACGGGCTCAGGCCGCGCCACCAGCAGCAGCACACA

**************************************************

T4g47970.2 GGGCGCGCCAGCTTTAGCGAGAGGGAGAGAGGGGGATTTTGGGCGGGGGA

T4g47970.1 GGGCGCGCCAGCTTTAGCGAGAGGGAGAGAGGGGGATTTTGGGCGGGGGA

**************************************************

T4g47970.2 GCGATCGATGGCGAAGCTGTACGTGCAGGCGGTGCAGCCGGCGGATCTGA

T4g47970.1 GCGATCGATGGCGAAGCTGTACGTGCAGGCGGTGCAGCCGGCGGATCTGA

**************************************************

T4g47970.2 ACAAGAACACGGAGTGGTTCATGTACCCCGGGGTGTGGACGACCTACATC

T4g47970.1 ACAAGAACACGGAGTGGTTCATGTACCCCGGGGTGTGGACGACCTACATC

**************************************************

T4g47970.2 CTCATCCTCTTCTTCTCCTGGCTGCTCGTCCTCTCCGTCTTCGGCTGCAC

T4g47970.1 CTCATCCTCTTCTTCTCCTGGCTGCTCGTCCTCTCCGTCTTCGGCTGCAC

**************************************************

T4g47970.2 CCCCGGCATGGCGTGGACGTTCGTCAACCTCGCCCACTTCGCGATGACAT

T 4g47970.1 CCCCGGCATGGCGTGGACGTTCGTCAACCTCGCCCACTTCGCGATGACAT

**************************************************

T4g47970.2 ACCATTTTTTTCACTGGAAGAAGGGAACTCCGTTTGCTGATGACCAGGGG

T 4g47970.1 ACCATTTTTTTCACTGGAAGAAGGGAACTCCGTTTGCTGATGACCAGGGG

**************************************************

T4g47970.2 ATGTATAATAGATTGACTTGGTGGGAGCAAATGGACAATGGGAAGCAGCT

T4g47970.1 ATGTATAATAGATTGACTTGGTGGGAGCAAATGGACAATGGGAAGCAGCT

**************************************************

T4g47970.2 TACTCGCAACAGAAAATTTCTGACCGTGGTTCCTTTGGTCCTGTAAG-AA

T4g47970.1 TACTCGCAACAGAAAATTTCTGACCGTGGTTCCTTTGGTCCTATACTTGA

****************************************** ** *

T4g47970.2 TAAATCTGT-TGCGATTGATTTTCCTTTTCACTACAGCATTGAAAATATG

T4g47970.1 TAGCCTTGCACACGACAGATTATCAACATCCTATGCTCTTCCTCAACACC

** ** *** **** ** ** * * ** *

T 4g47970.2 GATGATGAT-TGATTCTACATAGCCAGTAAGTAGTAACTAGTAAATGGTT

T 4g47970.1 ATTGCAGTTGTTGTGCTGGTTGTTGCAAAACTACCGAACATGCACAAGGT

** * * * * ** * ** ** * * * * *

T 4g47970.2 TCAGACTTTCAGTAGGTATGCAAGGAATTAACATATAGC--TCTGATGTT

T 4g47970.1 CCGGATCTTTGGAATCAATGCTGGCAACTAGGCGGCAGCATCCCACGGTT

* ** ** * * **** * ** ** *** * ***

T4g47970.2 TGTTACCGGCATAGT--CACA---GACTCACAGTAGATA-TACA------

T4g47970.1 TGTTATCACTACCATGCTTCATTTGAGTGGCAGGAAACAGTATACCTAGT

***** * * * ** ** * *** * * * ** *

T4g47970.2 --------------------------------------------------

T 4g47970.1 TCTGAATGGATAACCAGAGTGAGTAGTTTTTAAGAGCTGTTAGTAGATGA

T 4g47970.2 --------------------------------------------------

T4g47970.1 CATGTAATCATTGTACAGAGCTAAAGGCATGGAAATGATGTTGTTCAGCA

T4g47970.2 --------------------------------------------------

T4g47970.1 TCTAGTGAACACACTTCTTTAGGCTCTTATCCCACCAAAAAGAAACTTTA

T4g47970.2 --------------------------------------------------

T4g47970.1 TTTTGCTGTTAATGTTGTTATATATGTGCTCTTGTTCAAGTGTATGTCAC

T4g47970.2 --------------------------------------------------

T4g47970.1 ATGTGTGTCTTATGGAGAATAATTTTTTGTGTACGTAATGTGAGGCAGGA

T4g47970.2 --------------------------

T4g47970.1 AAGACAATATGCCTACTCATATCGCT

**Figure S2**. Sequence alignment of the two transcripts of Os04g47970.

The sequences were obtained from GRAMENE rice database.
