# Supplementary material for: Rice ORMDL Controls Sphingolipid Homeostasis Affecting Fertility Resulting from Abnormal Pollen Development
Source: PLoS One. 2014 Sep 5;9(9):e106386. doi: 10.1371/journal.pone.0106386 (PMC4156325; doi:10.1371/journal.pone.0106386)
Supplement: Figure S3 — Sequence alignment of the representative three transcripts of ORMDL genes. Highlight in yellow indicates sequences specific for Os07g26940. Highlight in gray indicates sequences used to make RNAi to knockdown all ORMDL genes. Green and red indicates primer sequences used for Os07g26940RNAi and ORMDL RNAi, respectively. The sequences were obtained from GRAMENE rice database. (DOC) [file pone.0106386.s003.doc]

T4g47970.1 GCGCCGTGCGGTGTGACGGCATTGGTGGCGCGGTGTGCGTCGCGGCACAC

T2g45180.1 --------------------------------------------------

t7g26940.1 --------------------------------------------------

T4g47970.1 AGCCGCACAGGCGCACAGCCAGGCAGCCAGCCCGTCGGTCTCCTCTCCTC

T2g45180.1 ----------------------------------------------GCTC

T7g26940.1 --------------------------------------------------

4g47970.1 TCCCCCTCGCGTTTTTTCTTTTCGCCTCCATCTTTGAACCTTCTCCTCGC

2g45180.1 TCACCGTCGCGTTATTTTTTCGC-CCCCCATCTTTGAACCAAACAGCCAA

7g26940.1 --------------------------------------------------

4g47970.1 CTTCTTCTTCCTCCTCCTCCCTCCGCTCCGCTTCTCTCTGACCAATAATC

2g45180.1 CCTCCTCCTCCTCCTCCTCCTCCTCCTCCTCGGCCTCGGCCTCGGTTCCT

7g26940.1 --------------------------------------------------

4g47970.1 TGGGCAAACCCCTCTC-TCGCGCCTTCTCCCTCCCACCTCTATAAATCCC

2g45180.1 CCTCCCCGCCGATCCCGCCGCTATAAATCCCTCCCAGCCGCGCGG---CT

7g26940.1 ----------GTACACAATAGGGTATCATCATCACACCATCACGAGCACC

* * * ** ** * *

4g47970.1 -CCTCCATTCCAATCCCACGGGCTCAGGCCGCGCCACCAGCAGCAGCAC-

2g45180.1 -CGGAGACGCGCTCGCCGCTCGCTCG---CTCGTCGCT-GCTCTGTTCC-

7g26940.1 ACCACCACTCACCACGCGCCACCCACCCCCGCACCGCACGCCAAGATCCG

* * * * * * * * * * ** *

4g47970.1 ACAGGGCGCGCCA--GCTTTAGCGAGAGGGAGAG-AGGGGGATTTTGGGC

2g45180.1 ACAACCGCCGGCG--GCCGGAGAGGGAGGGAAAGGAGAGGGAGGGAGGGG

7g26940.1 ACCGCCGCCGCCGCCGCCGCCGGAGTAGGGAGAGGGAGAGGAGATGGGGC

** ** * ** ***** ** *** ***

4g47970.1 GGGGGAGCGATCGATGGCGAAGCTGTACGTGCAGGCGGTGCAGCCGGCGG

2g45180.1 GGAGGCGGGGGCGATGGCGAAGCTGTACGTGCAGGCGGTGCCGCCGCCGG

7g26940.1 GGAGGGCGGGGTCCTACCCCTAC--TACGTGGAGGCGGCGCCGCCGGTGG

** ** * * * * ****** ****** ** **** **

4g47970.1 ATCTGAACAAGAACACGGAGTGGTTCATGTACCCCGGGGTGTGGACGACC

2g45180.1 ATCTGAACAGGAACACGGAGTGGTTCATGTACCCGGGCGTCTGGACGACC

7g26940.1 ACGTGAACAAGAACACGGAGTGGTTCATGTACCCCGGGGTGTGGACCACC

* ****** ************************ ** ** ***** ***

4g47970.1 TACATCCTCATCCTCTTCTTCTCCTGGCTGCTCGTCCTCTCCGTCTTCGG

2g45180.1 TACATCTGCATCCTCTTCTTCTCCTGGCTCCTCGTCCTCTCCGTCTTCGG

7g26940.1 TACATCCTCCTCCTCTTCTTCGCCTGGCTGCTCGTCCTCTCCGTCTCCGC

****** * *********** ******* **************** **

g47970.1 CTGCACCCCCGGCATGGCGTGGACGTTCGTCAACCTCGCCCACTTCGCGA

2g45180.1 CTGCACGCCCGGCATGGCCTGGACCGTCGTCAATCTCTTCCACTTCGCGA

7g26940.1 CTGCTCCCCCGGGATCGCGTGGACCGTCGTCAACCTCGCCCACTTCGCCA

**** * ***** ** ** ***** ******* *** ********* *

4g47970.1 TGACATACCATTTTTTTCACTGGAAGAAGGGAACTCCGTTTGCTG---AT

2g45180.1 TCACATACCACTTTTTCCATTGGAAGAAGGGAACACCTTTTGCTG---AT

7g26940.1 TCACTTATCACTTCTTCCATTGGAAGAAGGGAACTCCATTTGCTGCTGAT

* ** ** ** ** ** ** ************** ** ******* **

4g47970.1 GACCAGGGGATGTATAATAGATTGACTTGGTGGGAGCAAATGGACAATGG

2g45180.1 GACCAGGGAATGTACAACACATTGACTTGGTGGGAGCAAATGGACAATGG

7g26940.1 GACCAAGGCATCTACAACAGACTAACTTGGTGGGAACAAATTGATAACGG

***** ** ** ** ** * * * *********** ***** ** ** **

4g47970.1 GAAGCAGCTTACTCGCAACAGAAAATTTCTGACCGTGGTTCCTTTGGTCC

2g45180.1 CAAACAGCTTACTCGCAACAGGAAGTTCCTTGTTGTAGTTCCTGTTGTCC

7g26940.1 GCAGCAACTTACTCGTAATAGGAAGTTCTTGACTGTGGTACCTGTGGTGC

* ** ******** ** ** ** ** * ** ** *** * ** *

4g47970.1 TATACTTGATAGCCTTGCACACGACAGATTATCAACATCCTATGCTCTTC

2g45180.1 TGTATTTGATAGCTTCACACACTACAGACTACCAACATCCTATGCTCTTC

7g26940.1 TGTACCTGATCGCGTCACACTTGACCGACTACAAACAGCCAATGCTTTTC

* ** **** ** * *** ** ** ** **** ** ***** ***

4g47970.1 CTCAACACCATTGCAGTTGTTGTGCTGGTTGTTGCAAAACTACCGAACAT

2g45180.1 CTCAACACCCTTGCAGTCGCAGTGCTCGTGGTCGCTAAACTACCGAACAT

7g26940.1 CTCAACACCATTGCAGTTCTGGTACTAGTGGTAGCAAAGCTGCCAAACAT

********* ******* ** ** ** ** ** ** ** ** *****

4g47970.1 GCACAAGGTCCGGATCTTTGGAATCAATGCTGGCAACTAGGCGGCA-GCA

2g45180.1 GCACAAGGTCCGGATTTTTGGAATTAATGCTGGGAACTAGGCAGCA-GGA

7g26940.1 GCACAAGGTCCGTATATTTGGAATCAATGCAGATATCTGAGGACCATATG

************ ** ******** ***** * * ** * **

4g47970.1 TCCCACGGTTTGTTATCACTACCATGCTTCATTTGAGTGGCAGGAAACAG

2g45180.1 TCCTTCAGCA-GACATC--TACCTATCTTCCTTTC------------CAG

7g26940.1 AAGAACATCAGGGCACAA--GCTTCTGTGTGTTTGCAACG-AGGGACAAG

* * * * * * *** **

4g47970.1 TATACCTAGTTCTGAATGGATAACCAGAGTGAGTAGTTTTTAAGAGCTGT

2g45180.1 TTCATCTGGCTC--ATCAGAGTATCAAGG--AGTAGAGCTTAAATGTTGT

7g26940.1 CGAACTTTGTGCAAACACGGTATCTAAAGTAGTGAAAAAACTAAACTTTT

* * * * * * * * * * * * * *

4g47970.1 TAGTAGATGACATGTAATCATTGTACAGAG-----CTAAAGGCATGGAAA

2g45180.1 TAGCTGATGGGTTGTGATCGTTGTATAGAGAGATGCTCAGAGGCTGAAAA

7g26940.1 TCACCACTGCAATCTGTAGTATGTGCATAGGTGTAGGAAGAACCTGTAAA

* ** * * *** * ** * ** ***

4g47970.1 TGATGTTGTTCAGCATCTAGTGAACAC-ACTTCTTTAGGCTCTTA-TCCC

2g45180.1 CGATGCTGCTGTATATCTGATGTACACGAATTCTGTAGACTCTTA-ACGC

7g26940.1 TGTTACTGT--AGCTTATAATTTGCCTACAGGTTGGAGGCTGCTGCATGT

* * ** * * * * * ** ** *

4g47970.1 ACCAAAAAGAAACTTTATTTTGCTGTTAAT--GTTGTTATATATGTGCTC

2g45180.1 TGCTATATG--CTTTGATACAAATATCTGTCAGTTGCTGCATGTAGAGTC

7g26940.1 TTGCTCATGCCACTTCCTTTAAGAACTCT---AGTGGAGAGTGTAGACTG

* * ** * ** * * *

4g47970.1 TTGTT--CAAGTGTATG---TCACATGTGTGTCTTATGGAGAATAATTTT

2g45180.1 TTGTTTTCAGAACAATACCTTCATATACTTGTTTTCAGAACCATACTTGT

7g26940.1 TAACT--TAAATAAA------CATGTAATCTTGTGGATATGGAAAGTTTG

* * * * ** * * * * * **

4g47970.1 TTGTGTACGTAATGTGAGGCAGGAAAGACAATATGCCTACTCATATCGCT

2g45180.1 ATGGTTCTCCAAGATAGGAACACAGTGCGGGTCGCGTCAAACGCACAGGT

7g26940.1 GTGGTTGTTAAAATGGTGAACCGGAGTTCAGTTCCTGC------------

** * ** * *

4g47970.1 --------

2g45180.1 GATGGGAA

7g26940.1 --------

**Figure S3**. Sequence alignment of the representative three transcripts of *ORMDL* genes.

Highlight in yellow indicates sequences specific for Os07g26940. Highlight in gray indicates sequences used to make RNAi to knockdown all ORMDL genes. Green and red indicates primer sequences used for Os07g26940RNAi and ORMDL RNAi, respectively.

The sequences were obtained from GRAMENE rice database.
