# Supplementary material for: Rice ORMDL Controls Sphingolipid Homeostasis Affecting Fertility Resulting from Abnormal Pollen Development
Source: PLoS One. 2014 Sep 5;9(9):e106386. doi: 10.1371/journal.pone.0106386 (PMC4156325; doi:10.1371/journal.pone.0106386)
Supplement: Table S1 — qRT-PCR primer sequence. (DOC) [file pone.0106386.s004.doc]

**Table S1** qRT-PCR primer sequence

| Gene Name | Forward primer (5’-3’) | Reverse primer (5’-3’) |
| --- | --- | --- |
| Os05g36290(Actin1) | ATCCTTGTATGCTAGCGGTCGA | ATCCAACCGGAGGATAGCATG |
| Os03g08020(EF-1α ) | GTCATTGGCCACGTCGACTC | TGTTCATCTCAGCGGCTTCC |
| Os07g26930 | CCA AAA ACC GCT CAC ACT CG | GAA TTC GAT CCC AGG TGG CT |
| Os07g26940.1 | TGTGGTGCTGTACCTGATCG | CGGACCTTGTGCATGTTTGG |
| Os07g26940.2 | ACGGGCAGCAACTTACTCG | TGCTTCCACCCCAGAAATACT |
| Os07g26940.3 | CACTTCGCCGTACCTGATCG | CGGACCTTGTGCATGTTTGG |
| Os02g45180 | GCAGGATCCTTCAGCAGACA | ACTCTACATGCAGCAACTGACA |
| Os04g47970 | CCTCTTCTTCTCCTGGCTGCT | GTCCATTTGCTCCCACCAAGTC |
| Os02g56300 | GAGCTCTGTGATGAGTGGCA | CAAGGTAGTTTGCTGACGCA |
| Os03g14800 | GCGCTAAAGTTTCCATTCCG | GCTTCTTGGGCGGTTTGTAG |
| Os10g11200 | TATACCCGGGCTCGAAATTG | CCAGCTGACACGAATAAGCG |
| Os11g31640 | CGCATCTGCATTTCTGCTTC | AATCAACAACATGCGCCTTG |
| Os01g70380 | TGGTTGCAAGATTTGTTGGC | AACCCCTAGCCCCATTCACT |
| Os01g70370 | GCTATTCTCTTCGGCATGGG | CTGAACCCCTTGCTCCATTC |
| Os02g47350 | ATGGCATCAAATCCGGAAAG | CAAATTGCTGCAAATCGCAT |
| Os06g12250 | GGCATGACTCCTAAGACGGC | AGAAGGGCTGCGAGAAGTTG |
| Os02g51150 | CATGCGCTGTTCAACAACAA | CGTGACTCAAACCCTCCTCC |
| Os02g37080 | CATGATGCTAGGAACGCCAA | AGTGCGACCTCTCGTGCATA |
| Os02g49590 | ATCCCCTTATTCGCCGTCTT | CCAAAAGTTCCGCAGAAAGG |
| Os02g15750 | GACCCCGAGAACACCACATT | GTCCCTCTGCACCATCCTTC |
